# Spatiotemporal control of genome engineering in cone photoreceptors

**DOI:** 10.1186/s13578-023-01033-3

**Published:** 2023-06-28

**Authors:** Nan-Kai Wang, Pei-Kang Liu, Yang Kong, Yun-Ju Tseng, Laura A. Jenny, Nicholas D. Nolan, Nelson Chen, Hung-Hsi Wang, Chun Wei Hsu, Wan-Chun Huang, Janet R. Sparrow, Chyuan-Sheng Lin, Stephen H. Tsang

**Affiliations:** 1 Edward S. Harkness Eye Institute, Department of Ophthalmology, Columbia University Irving Medical Center, New York, NY 10032 USA; 2 Vagelos College of Physicians and Surgeons, Columbia University, New York, USA; 3 Department of Ophthalmology, Kaohsiung Medical University Hospital, Kaohsiung Medical University, Kaohsiung, Taiwan; 4 School of Medicine, College of Medicine, Kaohsiung Medical University, Kaohsiung, Taiwan; 5 Institute of Biomedical Sciences, National Sun Yat-sen University, Kaohsiung, Taiwan; 6 Department of Biomedical Engineering, The Fu Foundation School of Engineering and Applied Science, Columbia University, New York, NY 10027 USA; 7 Faculty of Health Sciences, Queen’s University, Kingston, ON Canada; 8 College of Arts and Sciences, University of Miami, Coral Gables, FL USA; 9 Departments of Ophthalmology, Pathology and Cell Biology, Columbia University, New York, USA; 10 Department of Pathology & Cell Biology, Columbia University Irving Medical Center, New York, NY 10032 USA; 11 Herbert Irving Comprehensive Cancer Center, Columbia University Irving Medical Center, New York, NY 10032 USA; 12 Jonas Children’s Vision Care, and Bernard and Shirlee Brown Glaucoma Laboratory, Columbia Stem Cell Initiative, Departments of Ophthalmology, Pathology and Cell Biology, Institute of Human Nutrition, Vagelos College of Physicians and Surgeons, Columbia University, New York, USA

**Keywords:** Cone arrestin, Arrestin 3, Cre-LoxP recombination, CreERT2, 2A peptide

## Abstract

**Background:**

Cones are essential for color recognition, high resolution, and central vision; therefore cone death causes blindness. Understanding the pathophysiology of each cell type in the retina is key to developing therapies for retinal diseases. However, studying the biology of cone cells in the rod-dominant mammalian retina is particularly challenging. In this study, we used a bacterial artificial chromosome (BAC) recombineering method to knock in the “CreER^T2^” sequence into the *Gnat2* and *Arr3* genes, respectively and generated three novel inducible CreER^T2^ mice with different cone cell specificities.

**Results:**

These models (*Gnat2*^*CreERT2*^, *Arr3*^*T2ACreERT2*^*, and Arr3*^*P2ACreERT2*^) express temporally controllable Cre recombinase that achieves conditional alleles in cone photoreceptors. Cre-LoxP recombination can be induced as early as postnatal day (PD) two upon tamoxifen injection at varying efficiencies, ranging from 10 to 15% in *Gnat2*^*CreERT2*^*,* 40% in *Arr3*^*T2ACreERT2*^, and 100% in *Arr3*^*P2ACreERT2*^. Notably, knocking in the P2A-CreERT2 cassette does not affect cone cell morphology and functionality. Most cone-phototransduction enzymes, including Opsins, CNGA3, etc. are not altered except for a reduction in the *Arr3* transcript.

**Conclusions:**

The *Arr3*^*P2ACreERT2*^ mouse, an inducible cone-specific Cre driver, is a valuable line in studying cone cell biology, function, as well as its relationship with rod and other retinal cells. Moreover, the Cre activity can be induced by delivering tamoxifen intragastrically as early as PD2, which will be useful for studying retinal development or in rapid degenerative mouse models.

**Supplementary Information:**

The online version contains supplementary material available at 10.1186/s13578-023-01033-3.

## Main text

### Background

Vision begins with the absorption of incoming light by the visual pigments in the outer segments of rod and cone photoreceptors in the retina. The photoreceptor cells receive light stimuli and are capable of converting light signals into electrical signals for vision formation in the cerebral cortex [1–3]. Photoreceptor cell death underlies both inherited and non-inherited retinopathies [4]. Of note, the cone photoreceptors, which are responsible for high-resolution vision and color discrimination [5, 6] are subject to cell death at different stages of degenerative retinopathies. Cone death is constantly identifiable at the preliminary stages of age-related macular degeneration (AMD) or cone-rod dystrophy [7] but in retinitis pigmentosa (RP) it is only notable at the final stage before total vision loss occurs. Therefore, it is cone death that causes blindness in many retinal diseases.

RP, the most common inherited retinal dystrophy, is associated with more than 150 genes identified thus far. Despite an overwhelming degeneration of rod photoreceptors, non-autonomous death of cone photoreceptors occurs in response to rod photoreceptor depletion in RP that is irrespective of underlying mutations [8–15]. Ceaseless efforts have been made to address potential interactions among retinal cell types that regulate cell death. Notably, a hypothesis about “metabolic coupling” has been proposed recently to explain different types of energy metabolism in photoreceptors (95% of the population are rods) and retinal pigment epithelium (RPE) in the vertebrate eye [16–18]. It primarily addresses the linkage in energy consumption between RPE and rod cells due to its abundance in the photoreceptor population [5]. The metabolic underpinnings explaining cone death in the setting of rod-specific mutations remain poorly understood [14]. The pathology of photoreceptors in AMD is better understood; outer and inner segment changes with mitochondrial translocations in macular cones indicate cone degeneration [7]. The density of mitochondria is different in rod photoreceptors compared to cone photoreceptors. Mitochondria in cones are twice as abundant as those in rods in rodents. In primates, the abundance of mitochondria in cones of primates is 10 times greater than that of rods [19, 20]. It has been demonstrated that in primary mitochondrial optic neuropathies, cone photoreceptors are more susceptible to mitochondrial dysfunction than rod photoreceptors [21]. This disparity suggests differential metabolic pathways in rod and cone photoreceptors.

Due to the differences in anatomical and physiological features between the rod and cone photoreceptors, and their delicate interactions with the other cell types in the retina, it is critical to investigate the pathophysiology of specific cell types to develop therapeutic interventions [11, 22–25]. Admittedly, detailed characterization of cone photoreceptors in the rod-dominant mammalian retina is particularly challenging [5]. A previous transgenic mouse model efficiently drives the expression of Cre recombinase in the cone photoreceptor by using human red-green pigment promoter (HRGP), which has been fundamental to studying cone photoreceptor gene functions [26]. However, this transgenic mouse line fails to induce the expression of Cre recombinase in a temporally-controllable manner [26]. In this study, we proposed three novel temporally-controllable cone-specific CreER^T2^ mice by knocking in the “CreER^T2^” sequence into *Gnat2* (G protein subunit alpha transducin 2, Gene ID: 14,686; MGI: 95,779) and *Arr3* (arrestin 3, retinal, Gene ID: 170735; MGI: 2159617, also known as arrestin 4 [ARR4], cone arrestin [CAR], X-arrestin; we use *Arr3* in this article) genes respectively by using bacterial artificial chromosome (BAC) recombineering. These models express temporally-controllable Cre recombinase upon tamoxifen induction to conditionally ablate flanked alleles by LoxP sites in a cone-specific manner. Unlike the conventional HRGP-Cre transgenic mice by random insertions, these models realize temporal regulation of the genes of interest in cone photoreceptors. The inducible manipulation of the alleles with cone-cell specificity in mouse models advances our understanding of the pathophysiology of cone photoreceptors and will be useful for developing therapeutic interventions against retinal degeneration. In contrast to the HRGP-Cre transgenic mice, our approach lacks the limitations inherent to the random integration of transgenes in conventional transgenesis. Moreover, scientists may use this approach to temporally investigate the in vivo functions of different genes in cone photoreceptors by crossing mice with different conditional floxed mice.

## Results

### Generation of *Gnat2*^*CreERT2*^ mice

**Fig. 1:**
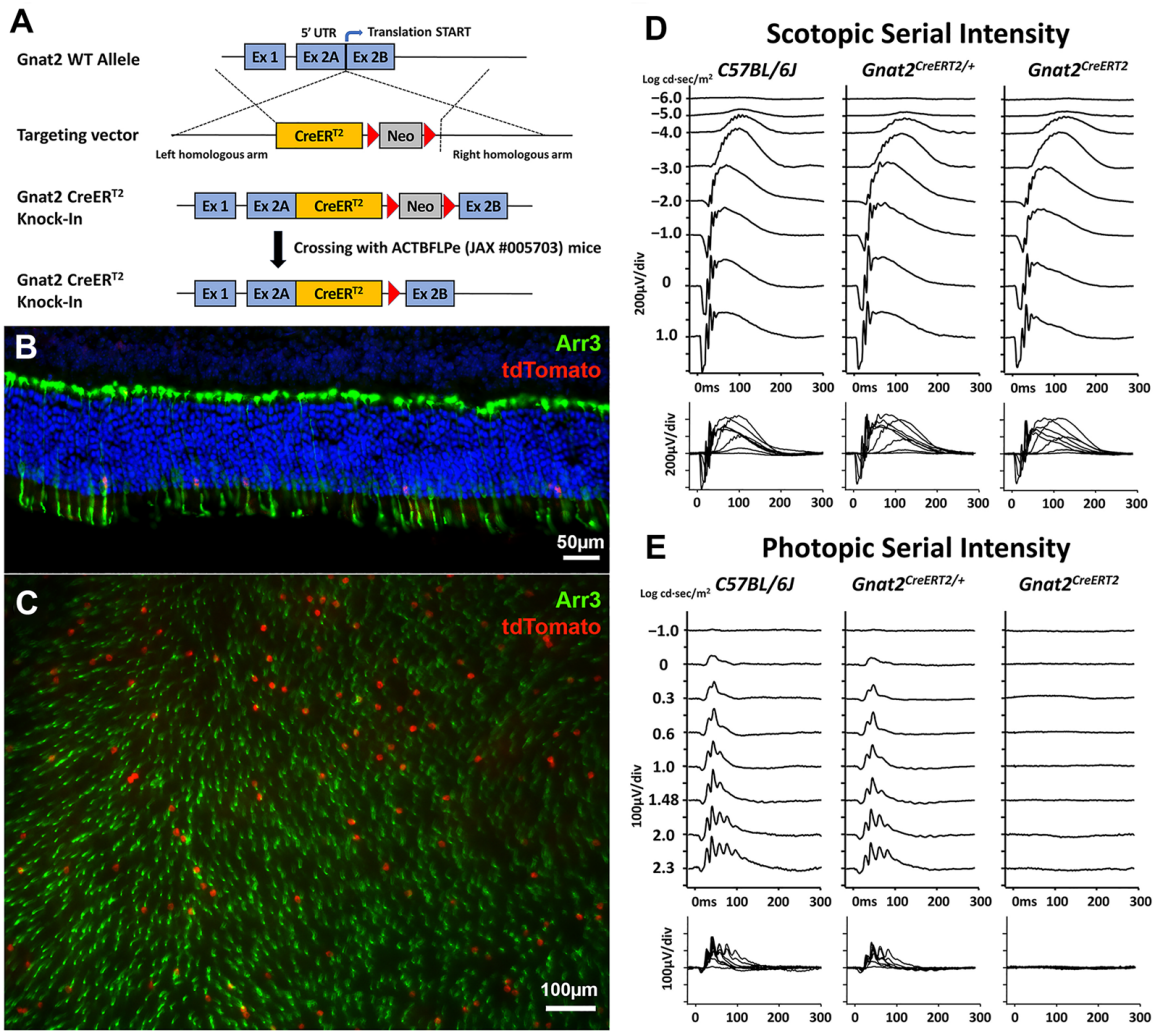
Generation, Cre-LoxP recombination efficiency and electroretinography (ERG) of inducible *Gnat2*^*CreERT2*^ mice. **A** Knock-in (KI) of a CreER^T2^ sequence to the translational start codon “ATG” in exon 2 of the *Gnat2* gene in mouse embryonic stem (ES) cells; confirmation of germline transmissions by crossing the male chimera with female *ACTB*-*FLPe* mice to remove the neomycin cassette. (**B** and **C**) Immunohistochemistry (IHC) of the retinal sections and whole mounts from 2-month-old *Gnat2*^*CreERT2/*+^*Ai14D*^*+/-*^ mice with an antibody against ARR3 (green). Around 10 to 15% of Arr3-positive cone cells (green) expressed td-Tomato (red) after tamoxifen induction. (**D** and **E**) Scotopic serial intensity ERG showed no significant difference in *a-* or *b-* wave amplitudes between *C57BL/6 J (wild-type, WT), Gnat2*^*CreERT2/*+^*, and Gnat2*^*CreERT2*^ mice at 2 months-old; photopic serial intensity ERG showed no significant difference between WT*, Gnat2*^*CreERT2/*+^ mice, however, there were extinguished responses in *Gnat2*^*CreERT2*^ mice, which indicated a loss of cone function in homozygous *Gnat2*^*CreERT2*^ mouse

In order to generate an inducible cone-specific Cre mouse line, we engineered a mouse model with the BAC recombineering method to introduce a CreER^T2^ sequence into the translational start codon “ATG” in exon 2 of the *Gnat2* gene in mouse embryonic stem (ES) cells. Germline transmissions were confirmed after crossing the male chimeric mice with female B6.Cg-Tg(ACTFLPe)9205Dym/J (JAX #005703, hereafter *ACTB*-*FLPe*) mice to remove a neomycin cassette (Fig. 1A). The first filial generation hybrid offspring (F1) *Gnat2*^*CreERT2/*+^ mice were then intercrossed to generate *Gnat2*^*CreERT2*^ mice.

### *The specificity and efficiency of Cre-LoxP recombination in Gnat2*^*CreERT2*^* mice*

To assess Cre-LoxP recombination specificity and efficiency, homozygous *Gnat2*^*CreERT2*^ mice were crossed to the B6.Cg-Gt(ROSA)26Sor^tm14(CAG−tdTomato)Hze^/J (JAX #007914, hereafter *Ai14D*) reporter mice, known for expressing tdTomato fluorescence following Cre-mediated recombination. Tamoxifen was injected intraperitoneally at 100 μg/g of the body weight into the *Gnat2*^*CreERT2/*+^*Ai14D*^*+/-*^ mice at postnatal day (PD) 21 for 3 consecutive days. Seven days after tamoxifen induction, Cre-mediated recombination was assessed via cryosection immunohistochemistry (IHC) and retinal whole mounts. The number of tdTomato-positive cells, based on our observation, is 10 to 15% of ARR3-positive cone cells (Fig. 1B and C). IHC on retinal section with Cre recombinase showed 100% colocalization of Cre recombinase and td-Tomato (Additional file 1: Fig. S3 A, B).

### *Impact of the Knock-In (KI) CreER*^*T2*^* cassette on cone function in Gnat2*^*CreERT2*^* mice*

To further examine whether cone cell function was affected by replacing the translational ATG start codon of the *Gnat2* gene with CreER^T2^ cassette, *Gnat2*^*CreERT2/*+^ mice were intercrossed to generate *C57BL/6 J (*JAX #000664, hereafter wild-type, WT*), Gnat2*^*CreERT2/*+^*, and Gnat2*^*CreERT2*^ mice. Electroretinography (ERG) was performed on these littermates. Scotopic serial intensity ERG showed no significant difference in *a-* and *b-*wave amplitudes among the WT*, Gnat2*^*CreERT2/*+^ *and Gnat2*^*CreERT2*^ mice at two month of age; photopic serial intensity ERG showed no significant difference between WT*, Gnat2*^*CreERT2/*+^ mice. However, extinguished photopic responses in *Gnat2*^*CreERT2*^ mice were noted, indicating a loss of cone function in *Gnat2*^*CreERT2*^ mice (Fig. 1D, E). We speculated that replacing the translational ATG start codon of the *Gnat2* gene with the CreERT2 cassette disrupts GNAT2 function, which phenotypically mimics the *Gnat2*^*cpfl3*^ mice (JAX #006795) [27].

### Generation of *Arr3*^*T2ACreERT2*^ mice

**Fig. 2:**
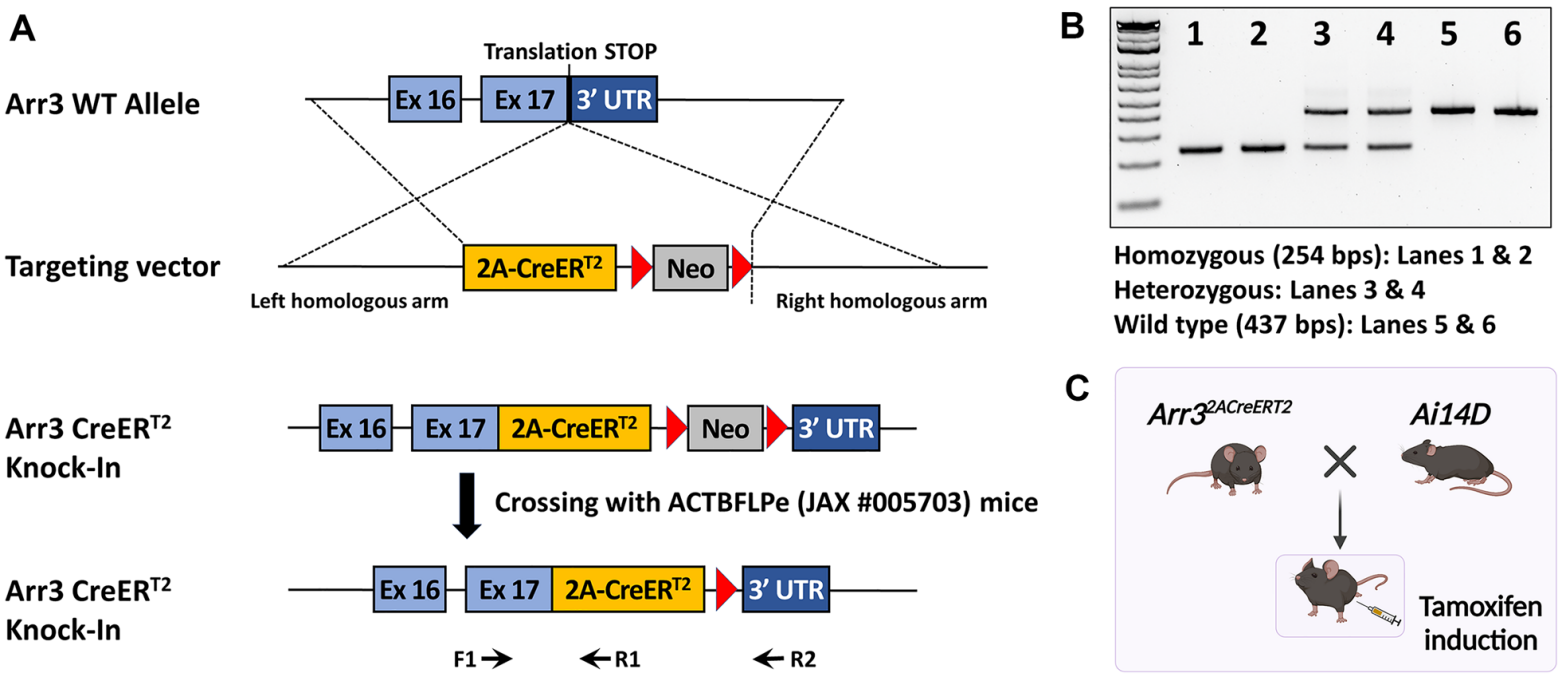
Generation of Arr3^2ACreERT2^ driver. **A** KI of a 2A peptide with the CreER^T2^ sequence to the stop codon “TAG” of *Arr3* gene in exon 17 in mouse embryonic stem (ES) cells; confirmation of germline transmissions by crossing the male chimera with female *ACTB*-*FLPe* mice to remove the neomycin cassette; genotyping for the KI allele by primers F1 and R1 and for the wild type (WT) allele by the primers F1 and R2. **B** Electrophoresis of the amplicons of the KI allele (~ 254 bp) and the WT allele (~ 437 bp). **C** Schematic representation of assessing recombination specificity and efficiency of the Cre-LoxP: a female homozygous *Arr3*^*2ACreERT2*^ mouse was crossed with a male *Ai14D* reporter mouse to generate male hemizygous *Arr3*^*2ACreERT2*^*Ai14D*^*+/-*^ and female heterozygous *Arr3*^*2ACreERT2/+*^*Ai14D*^*+/-*^ mice

Because the Cre-LoxP recombination efficiency was 10% in our *Gnat2*^*CreERT2*^ mice, we chose the *Arr3* gene, which is cone-specific and expressed at embryonic day E 15.5 [28]. In addition, we modified our approach in targeting embryonic stem (ES) cells by introducing a 2A peptide and CreER^T2^ cassette into the translational “STOP” codon of the *Arr3* gene to avoid disruption of the upstream gene (Fig. 2A). As an alternative to replacing the translational “START” codon of *Gnat2* with the CreERT2 cassette, we hypothesized that introducing the “T2A-CreER^T2^” into the translational STOP codon (Fig. 2A) ensures expression of both ARR3 and the downstream CreER^T2^. This strategy avoids functional perturbation of cone cells as observed in homozygous *Gnat2*^*CreERT2*^ mice. Genotypes were determined by amplifying the KI allele (primers F1&R1) and WT allele (primers F1 and R2) (Fig. 2B). Germline transmission was confirmed by crossing the male chimeric mice to female *ACTB*-*FLPe* mice to remove the neomycin cassette. Male hemizygous mice were intercrossed with female heterozygous *Arr3*^*T2ACreERT2/*+^ mice to generate WT, male hemizygous, female heterozygous and homozygous mice for the ensuing experiments. All the mice were viable, fertile, normal in size and do not display any gross physical or behavioral abnormalities.

### *The specificity and efficiency of Cre-LoxP recombination in Arr3*^*T2ACreERT2*^* mice*

**Fig. 3:**
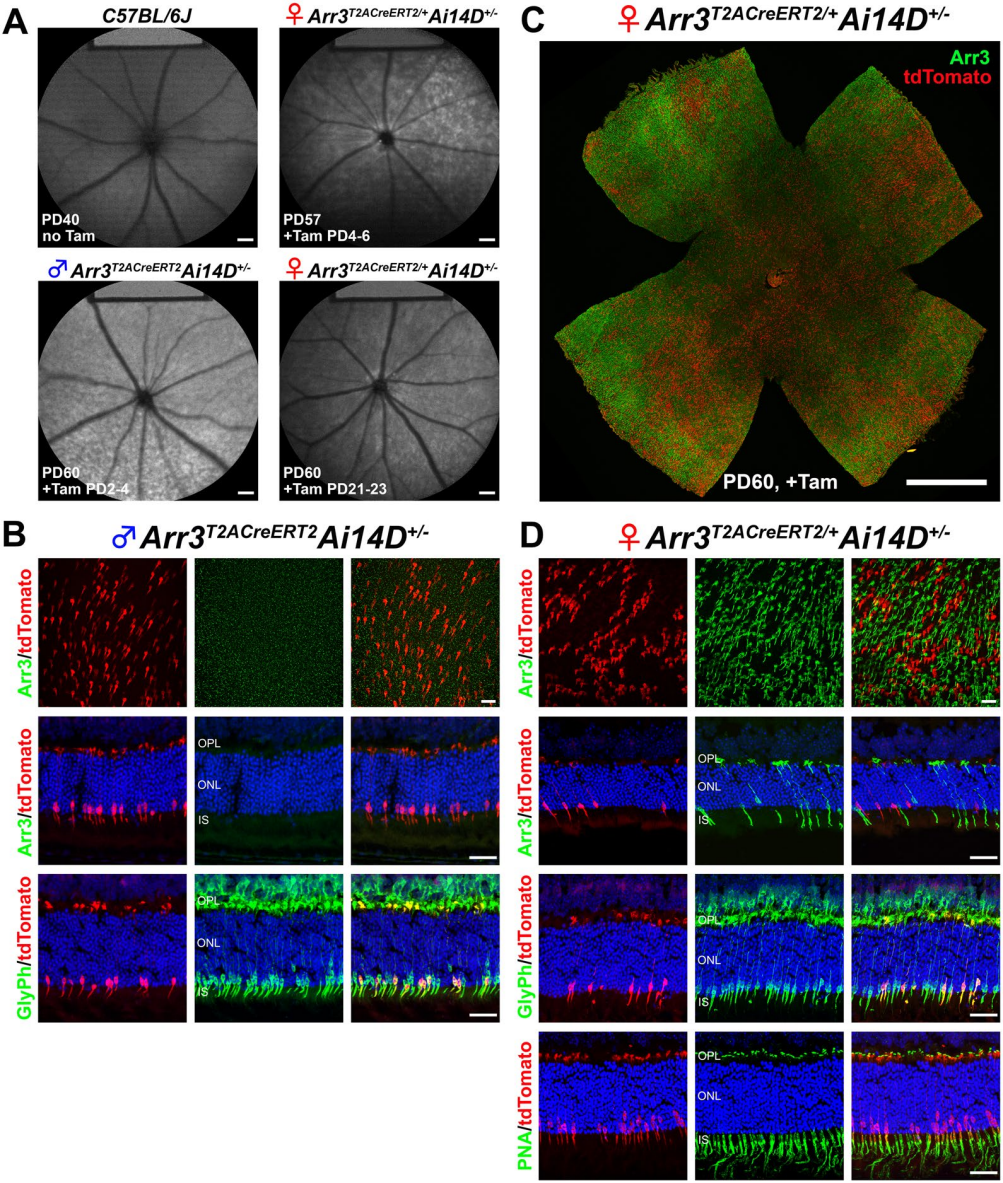
Cone-specific Cre-LoxP recombination activity in *Arr3*^*T2ACreERT2*^ mouse retina. **A** Noninvasive in vivo fundus short-wavelength autofluorescence (SW-AF) showed a homogenous background in the *C57BL/6J* mouse, increased intensity, and a “mosaic” pattern of SW-AF in male hemizygous *Arr3*^*T2ACreERT2*^*Ai14D*^*+/-*^ and female heterozygous *Arr3*^*T2ACreERT2/+*^*Ai14D*^*+/-*^ mice. There was no difference in the intensity or mosaicism of SW-AF images when tamoxifen induction was delivered intragastrically at postnatal day (PD) 4–6 or intraperitoneally at PD21-24. **B** Immunohistochemistry (IHC) staining on retinal whole mounts and crysections from male hemizygous *Arr3*^*T2ACreERT2*^*Ai14D*^*+/-*^ mice showed distinct tdTomato signal in cone cells (red) that did not co-label with anti-ARR3 antibody staining (green) (1st and 2nd rows; Additional file 1: Movie S1). When the same sections were labeled for another cone cell marker, glycogen phosphorylase (GlyPh), we found that 40% of GlyPh-positive cones also expressed tdTomato (bottom; Additional file 1: Movie S2), suggesting that the Cre-LoxP recombination efficiency was 40% and not every cone cell in hemizygous *Arr3*^*T2ACreERT2*^*Ai14D*^*+/-*^^±^ mice expresses tdTomato. **C**–**D** IHC staining for the anti-ARR3 antibody on a retinal whole mount and cryosections from a female heterozygous *Arr3*^*T2ACreERT2/+*^*Ai14D*^*+/-*^ mouse showed a mosaic pattern of green (ARR3-positive cone cells) and red (tdTomato expressing cone cells) in retinal whole mounts and cryosections (1st and 2nd rows; Additional file 1: Movies S3, S4). IHC staining for anti-GlyPh and PNA antibodies on retinal cryosections from a female heterozygous *Arr3*^*T2ACreERT2/+*^*Ai14D*^*+/-*^ mouse showed around 50% of GlyPh-positive or PNA-positive cone cells express tdTomato (*3*^*rd*^* and 4*^*th*^* rows;* Additional file 1: Movie S5). *PD* postnatal day, *Tam* tamoxifen, *OPL* outer plexiform layer, *ONL* outer nuclear layer, *IS* inner segment, Ai14D: B6.Cg-Gt(ROSA)26Sor^tm14(CAG−tdTomato)Hze^/J (JAX #007914). Scale bars, 200 μm in **A**, 20 μm in **B** and **D**, and 1000 μm in **C**

To assess the specificity and efficiency of Cre-LoxP recombination, female homozygous *Arr3*^*T2ACreERT2*^ mice were crossed to the male *Ai14D* reporter mice to generate male hemizygous *Arr3*^*T2ACreERT2*^*Ai14D*^*+/-*^ and female heterozygous *Arr3*^*T2ACreERT2/+*^*Ai14D*^*+/-*^ mice. Considering that the *Arr3* gene is located on the X-chromosome, we examined the X-chromosome inactivation (XCI) (or “lyonization”, after geneticist Mary Lyon, who first proposed such mechanism to occur) [29] by using noninvasive in vivo fundus short-wavelength autofluorescence (SW-AF) on *Arr3*^*T2ACreERT2/+*^*Ai14D*^*+/-*^ mice. The red fluorescent protein tdTomato in cone cells increases autofluorescence on SW-AF. After tamoxifen induction, we observed increased intensity and mosaicism of SW-AF in both male hemizygous *Arr3*^*T2ACreERT2*^*Ai14D*^*+/-*^ and female heterozygous *Arr3*^*T2ACreERT2/+*^*Ai14D*^*+/-*^ mice at PD40, compared to the WT (Fig. 3A, internal fluorescent reference on the top of SW-AF image) [30]. To optimize the expression of Cre recombinase, SW-AF was carried out on the mice subject to tamoxifen induction at PD2-4, and PD21-23, respectively. No difference in intensity or mosaicism was identified (Fig. 3A*,* right panels), suggesting that Cre-LoxP recombination could be induced as early as PD2 despite not all the cone photoreceptors expressing tdTomato.

We stained retinal whole mounts from mice described above for the cone marker (anti-ARR3 antibody) and retinal cryosections for cone markers. In male hemizygous *Arr3*^*T2ACreERT2*^*Ai14D*^*+/-*^ mice, we observed distinct tdTomato expression in cone cells; however, the tdTomato signal did not co-localize with the ARR3-labeling (in green). These unexpected findings are consistent in retinal whole mounts and cryosections (Fig. 3B, 1st and 2nd rows; Additional file 1: Movie S1). To confirm the presence of the cone photoreceptors, glycogen phosphorylase (GlyPh), another cone-specific marker labeling mouse cone cells from their pedicles to outer segments, was employed [31] (1:500; kind gift from Dr. Pfeiffer-Guglielmi, University of Tübingen, Tübingen, Germany). Based on our observations, forty percent of GlyPh-positive cone cells co-express tdTomato (Fig. 3B, bottom rows; Additional file 1: Movie S2), suggesting a 40% recombination efficiency of the Cre-LoxP. Furthermore, we noted that not every cone cell expresses tdTomato in hemizygous *Arr3*^*T2ACreERT2*^*Ai14D*^*+/-*^ mice, which may account for the mosaic SW-AF phenotype in the male hemizygous *Arr3*^*T2ACreERT2*^*Ai14D*^*+/-*^ mice (Fig. 3A). In female heterozygous *Arr3*^*T2ACreERT2/+*^*Ai14D*^*+/-*^ mice, we found a mosaic of green (ARR3-positive cone cells) and red (tdTomato expressing cone cells) fluorescence in retinal whole mounts and cryosections (Fig. 3C, D, 1st and 2nd rows). It is worth noting that green- and red-labelled cone cells are not co-localized (Additional file 1: Movies S3, S4). Further verification by using GlyPh and peanut agglutinin (PNA) showed that 40 to 50% of GlyPh-positive or PNA-positive cone cells co-express tdTomato (Fig. 3D, 3rd and 4th rows; Additional file 1: Movie S5). Taken together, the IHC staining results from male hemizygous and female heterozygous mouse retina indicate that tdTomato-positive cone cells were faintly labeled with the anti-ARR3 antibody (Green). Moreover, 40% of cone cells with active KI *Arr3*^*T2ACreERT2*^ X-chromosomes in male hemizygous mice express tdTomato indicating the efficiency of Cre-LoxP recombination is 40% in male *Arr3*^*T2ACreERT2*^ mice.

### *Impact of the KI T2A-CreER*^*T2*^* cassette on ARR3 protein expression and cone function in Arr3*^*T2ACreERT2*^* mice*

**Fig. 4:**
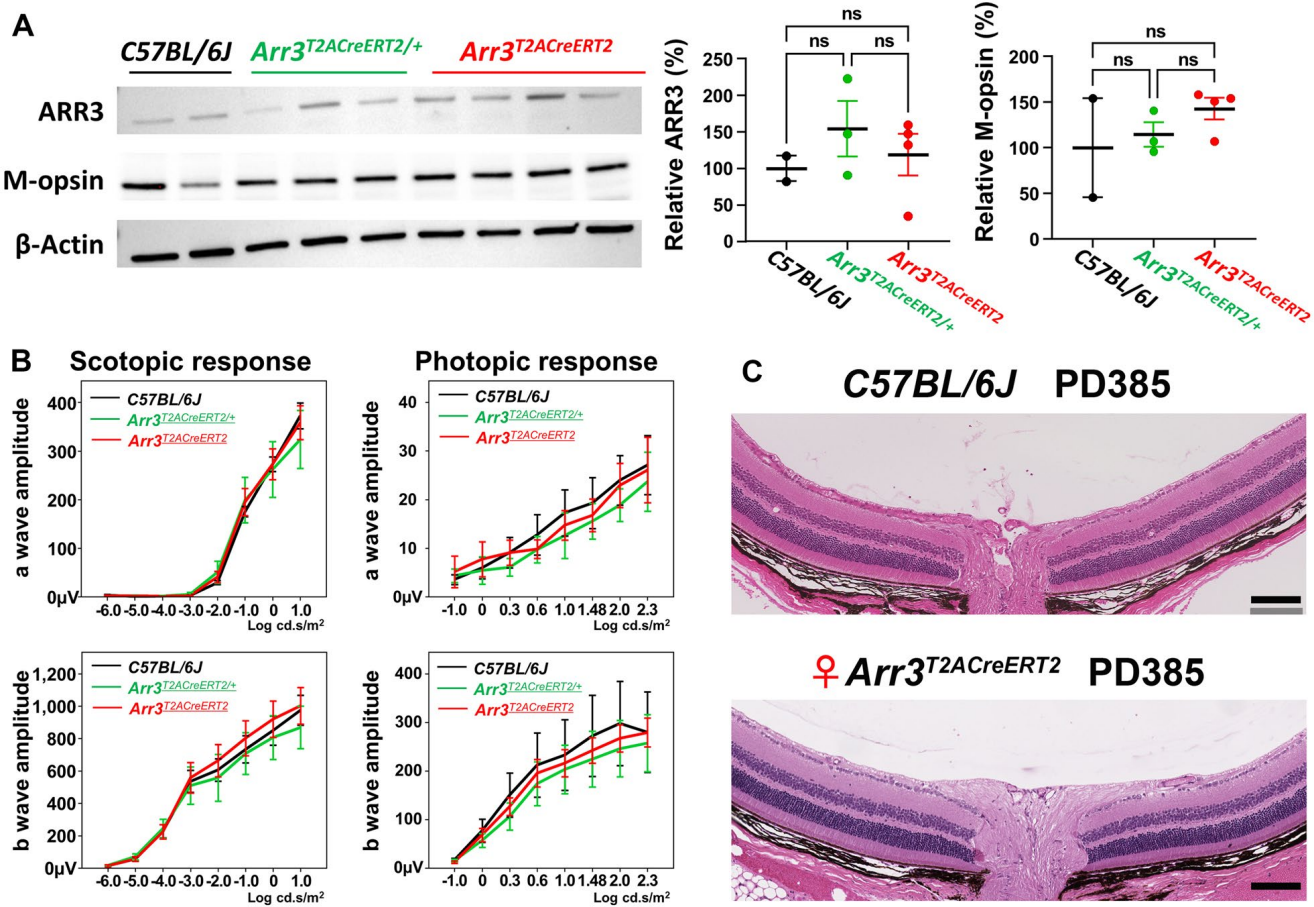
Impact of KI T2A-CreER^T2^ cassette on retinal protein and function in *Arr3*^*T2ACreERT2*^ driver. **A** Immunoblot of retina lysates from mice at PD60 using anti-ARR3 and anti-M-opsin antibodies revealed comparable protein expression in WT, heterozygous, and homozygous *Arr3*^*T2ACreERT2*^ mice quantified by ARR3 and M-opsin on the right. *F*(2, 6) = 0.616, *p* = 0.570; *F*(2,6) = 0.964, *p* = 0.433, respectively (One-way ANOVA). *N* = 2, 3, and 4 mice for WT, female heterozygous, and female homozygous *Arr3*^*T2ACreERT2*^ mice, respectively. **B** Scotopic serial intensity ERG showed no significant difference in *a-* or *b-* wave amplitudes between mice of three different genotypes. (One-way ANOVA, Additional file 1: Table S3). N = 4, 4, and 7 mice for WT, female heterozygous, and female homozygous *Arr3*^*T2ACreERT2*^ mice, respectively. **C** Histology showed normal retinal thickness and outer nuclear layers at PD385 homozygous *Arr3*^*T2ACreERT2*^ and age-matched WT mice. Data represent mean ± 2SE in *A* and *B*

The anti-ARR3 antibody used above recognizes an epitope within 12 amino acids at the C-terminus of ARR3 protein. We hypothesize that T2A alters the C-terminus of the ARR3 protein, and causes the cones to be unlabeled by the anti-ARR3 antibody in IHC (Fig. 3B, 1st and 2nd rows; Additional file 1: Movie S1). Thus, we immunoblotted retina lysates using the same anti-ARR3 antibody in IHC staining to examine the ARR3 protein expression in the retina. No significant reduction in ARR3 expression in homozygous mice compared to WT and heterozygous mice was observed (*F*_2, 6_ = 0.616, *p* = 0.570) (Fig. 4A). Thus, the results from immunoblotting suggest that the anti-ARR3 antibody could recognize the denatured T2A modified ARR3 protein even though the same anti-ARR3 antibody could not recognize the same protein when it retained its tertiary structure in IHC staining. To further test whether the KI T2A-CreER^T2^ cassette could affect other cone-specific proteins which interact with ARR3, we performed immunobloting with an anti-M-opsin antibody, which showed no significant difference between mice of different genotypes, *F*_2, 6_ = 0.964, *p* = 0.433 (Fig. 4A). (*N* = 2, 3, and 4 mice for WT, heterozygous, and female homozygous *Arr3*^*T2ACreERT2*^ mice, respectively).

It has been demonstrated that in mice having a cone arrestin knockout (*Arr4*^*−/−*^), the photopic *a-* and *b-* wave amplitudes in young mice (defined as 2 months old) were significantly increased compared to age-matched WT mice [32]. Although there was no significant decrease in ARR3 protein expression in our *Arr3*^*T2ACreERT2*^ mouse based on immunoblotting analysis, it remains unknown whether KI T2A-CreER^T2^ cassette at the C-terminus of ARR3 would affect its role in the shutoff mechanism in the cone phototransduction cascade. The latter would result in increased photopic ERG amplitudes because the C-terminus domain of ARR3 protein consists of an immunoglobulin-like β- sandwich structure, which could bind to phosphorylated red/green opsins and shutoff the cone phototransduction cascade [33]. To test this hypothesis, scotopic and photopic electroretinograms (ERG) were recorded from litter control WT, heterozygous, and homozygous mice at an age of 2 months. The *a-* and *b-* wave amplitudes amongst the three different genotypes were comparable (Fig. 4B). Histology of PD385 mouse retina showed comparable thickness of the outer nuclear layer between WT and *Arr3*^*T2ACreERT2*^ mice (Fig. 4C), demonstrating an intact retinal morphology in the presence of the KI T2A-CreER^T2^ cassette.

### Generation of *Arr3*^*P2ACreERT2*^ mice

We found a 40% efficiency of Cre-LoxP recombination in male hemizygous *Arr3*^*T2ACreERT2*^ mice, and cone cells that express active KI *Arr3*^*T2ACreERT2*^ X-chromosomes could only be spottily labeled with anti-ARR3 antibody. To test whether these unexpected results in *Arr3*^*T2ACreERT2*^ mice could be improved by replacing “P2A” with “T2A” peptide, we engineered an *Arr3*^*P2ACreERT2*^ mouse using the same method as the *Arr3*^*T2ACreERT2*^ mouse, except the “T2A-CreER^T2^” sequence was changed to “P2A-CreER^T2^”. All these mice were fully viable, fertile, normal in size, and did not display any gross physical or behavioral abnormalities.

### *Cre-LoxP recombination specificity and efficiency of Arr3*^*P2ACreERT2*^* mice*

**Fig. 5:**
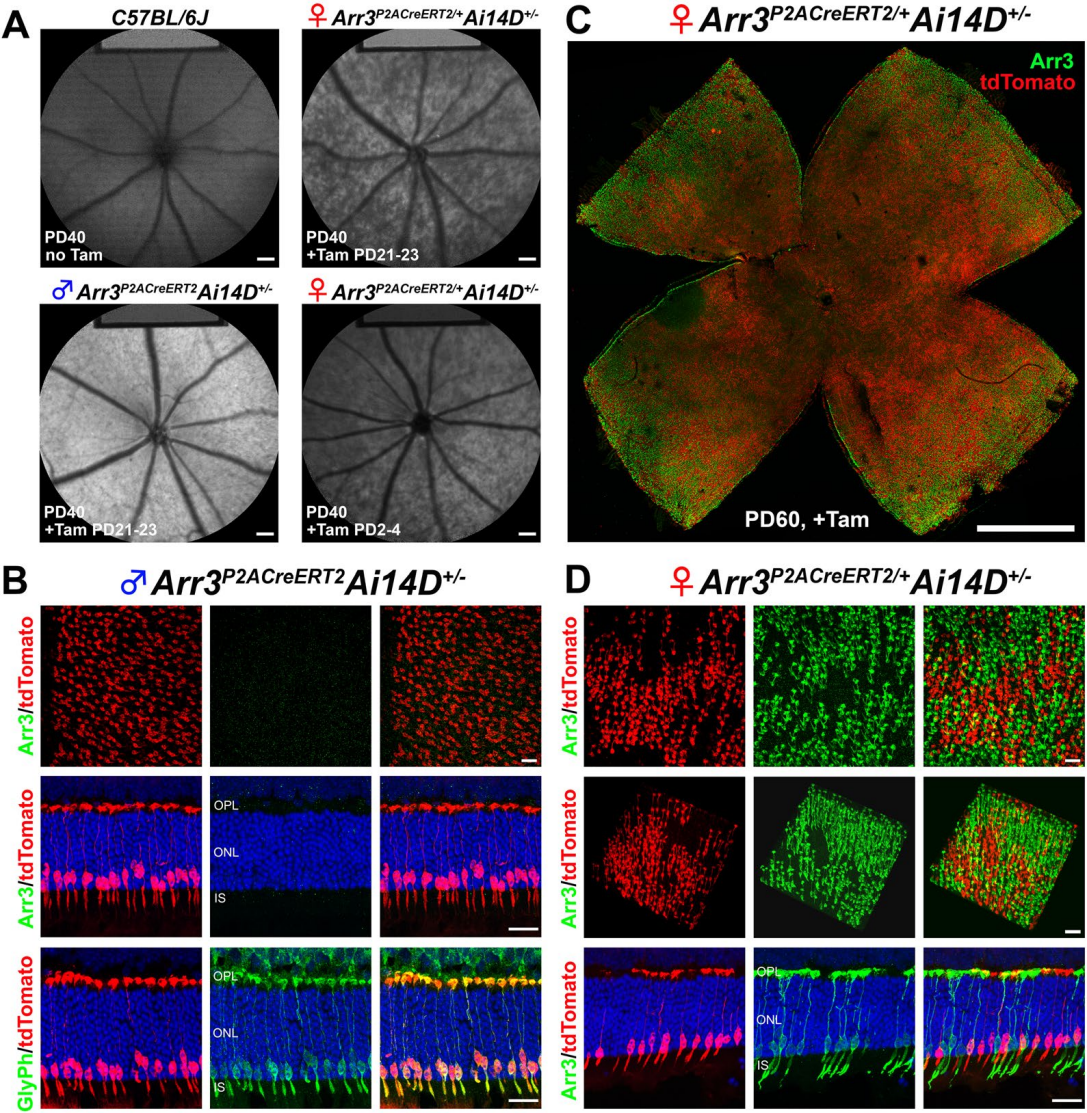
Cre-LoxP recombination efficiency was 100% in hemizygous *Arr3*^*P2ACreERT2*^ driver. **A** Noninvasive in vivo fundus short-wavelength autofluorescence (SW-AF) showed homogenously increased intensity in male hemizygous *Arr3*^*P2ACreERT2*^*Ai14D*^*+/-*^ compared to the SW-AF in WT (*C57BL/6 J*) mice. In female heterozygous *Arr3*^*P2ACreERT2/+*^*Ai14D*^*+/-*^ mice, there was a mosaic pattern of SW-AF images when tamoxifen induction was delivered at PD21-24 or PD2-4 (*upper right and lower right*). **B** Immunohistochemistry (IHC) staining on a retinal whole mount and crysections from male hemizygous *Arr3*^*P2ACreERT2*^*Ai14D*^*+/-*^ mice showed distinct tdTomato expression in cone cells with ARR3 barely labeled in green (1st and 2nd rows; Additional file 1: Movie S6, S7). When the same sections were labeled for another cone cell marker, glycogen phosphorylase (GlyPh), we found 100% colocalization of GlyPh (green) and tdTomato (red) (*bottom;* Additional file 1: Movie S8), suggesting that the Cre-LoxP recombination efficiency was 100% in hemizygous *Arr3*^*P2ACreERT2*^*Ai14D*^*+/-*^ mice. **C–D** IHC staining for the anti-ARR3 antibody on a retinal whole mount and cryosections from a female heterozygous *Arr3*^*P2ACreERT2/+*^*Ai14D*^*+/-*^ mouse showed a mosaic pattern of green (ARR3-positive cone cells) and red (tdTomato expressing cone cells) in retinal whole mounts and cryosections (1st and 2nd rows; Additional file 1: Movies S9, S10). Scale bars, 200 μm in **A**, 20 μm in **B **and **D**, and 1000 μm in **C**

To assess the specificity and efficiency of Cre-LoxP recombination, we crossed the *Arr3*^*P2ACreERT2*^ mice with *Ai14D* reporter mice. After tamoxifen induction at PD 21–23, male hemizygous *Arr3*^*P2ACreERT2*^*Ai14D*^*+/-*^ mice displayed homogeneous SW-AF with greater intensity (Fig. 5A, lower left) compared to the *B6* mice at PD40 (Fig. 5A, upper left). It is worth noting that the SW-AF in male hemizygous *Arr3*^*P2ACreERT2*^*Ai14D*^*+/-*^ mice are more homogenously brighter than the “mosaic” pattern of SW-AF in male hemizygous *Arr3*^*T2ACreERT2*^*Ai14D*^*+/-*^ mice (Fig. 3A, lower left). In female heterozygous *Arr3*^*P2ACreERT2/+*^*Ai14D*^*+/-*^ mice, we observed mosaic-like phenotypes in SW-AF images (Fig. 5A, upper right and lower right) that was similar to the SW-AF patterns in female heterozygous *Arr3*^*T2ACreERT2/+*^*Ai14D*^*+/-*^ mice, indicating that not all the cone cells express tdTomato fluorescence due to random XCI. The mosaic could be observed in SW-AF images of female heterozygous *Arr3*^*P2ACreERT2/+*^*Ai14D*^*+/-*^ mice when tamoxifen was delivered at PD2-4 through intragastric injections (Fig. 3A, lower right).

Retinal whole mounts and cryosections collected from the mice described above were stained for cone markers. In male hemizygous *Arr3*^*P2ACreERT2*^*Ai14D*^*+/-*^ mice, we observed distinct tdTomato expression in cone cells and very faintly labeled ARR3 as green signal (Fig. 5B, 1st and 2nd row; Additional file 1: Movies S6, S7). The tdTomato-positive cone cells are much denser in hemizygous *Arr3*^*P2ACreERT2*^*Ai14D*^*+/-*^ mice (Fig. 5B, 1st row) than in hemizygous *Arr3*^*T2ACreERT2*^*Ai14D*^*+/-*^ mice (Fig. 3B, 1st rows). GlyPh staining revealed 100% co-localization of GlyPh with tdTomato (Fig. 5B, bottom rows; Additional file 1: Movie S8) whereas only 40% co-localization of GlyPh with tdTomato was identified in the hemizygous *Arr3*^*T2ACreERT2*^*Ai14D*^*+/-*^ mice (Fig. 3B, bottom rows; Additional file 1: Movie S2). These results indicate 100% efficiency of Cre-LoxP recombination in the presence of a P2A-CreER^T2^ cassette. Every cone cell in hemizygous *Arr3*^*P2ACreERT2*^*Ai14D*^*+/-*^ mice expresses tdTomato, which reveals the absence of SW-AF mosaicism in male hemizygous *Arr3*^*P2ACreERT2*^*Ai14D*^*+/-*^ mice (Fig. 5A, lower left). In female heterozygous *Arr3*^*P2ACreERT2/+*^*Ai14D*^*+/-*^ mice, we found a mosaic pattern of green (ARR3-positive cone cells) and red (tdTomato from active KI *Arr3*^*P2ACreERT2*^ allele cone cells) in retinal whole mount retina and cryosections (Fig. 5C, D, 1st and 2nd rows). Similar to *Arr3*^*T2ACreERT2/+*^*Ai14D*^*+/-*^ mice, green- and red- labelled cones are not co-localized (Additional file 1: Movies S9, S10). Additionally, 5 month-old delayed tamoxifen induction in male hemizygous *Arr3*^*P2ACreERT2*^*Ai14D*^*+/-*^ mice revealed a homogenous, bright SW-AF presentation (Additional file 1: Fig. S4A). Flat-mounted retinas revealed co-localized anti-PNA and tdTomato, indicating robust cone-specific recombination (Additional file 1: Fig. S4C, D). Glyph, Arr3, and PNA presented similarly in stained, retinal sections as earlier time-point induced *Arr3*^*P2ACreERT2*^*Ai14D*^*+/-*^ mice (Additional file 1: Fig. S4B). Taken together the IHC staining results from male hemizygous and female heterozygous *Arr3*^*P2ACreERT2/+*^ mouse retina indicate that cone cells with an active KI *Arr3*^*P2ACreERT2*^ X-chromosome can efficiently express tdTomato via Cre-LoxP recombination; however, these tdTomato-positive cone cells were faintly labeled with an anti-ARR3 antibody, which is similar to the *Arr3*^*T2ACreERT2*^ mouse.

### *Impact of the KI P2A-CreER*^*T2*^* cassette on Cone protein and gene expression in Arr3*^*P2ACreERT2*^* mice*

**Fig. 6:**
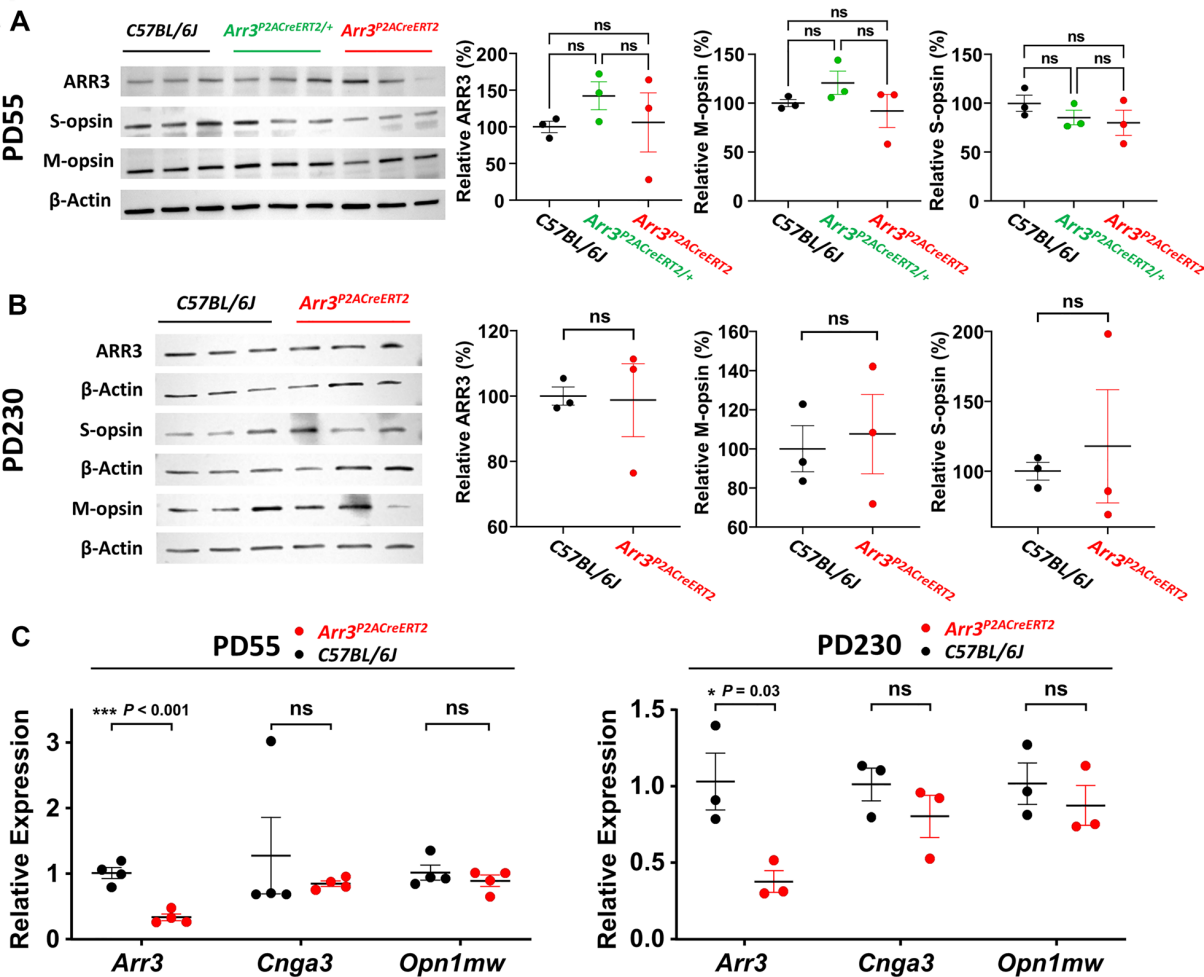
Comparable ARR3 protein expression in *Arr3*^*P2ACreERT2*^ mice and B6 controls. **A** Immunoblot of retinal lysates collected from mice at PD55 involved in cone phototransduction shows comparable protein expression in WT, heterozygous, and homozygous *Arr3*^*P2ACreERT2*^ mice, quantified by ARR3, M-opsin, and S-opsin from left to right. *F*(2, 6) = 0.756, *p* = 0.510; *F*(2,6) = 1.514, *p* = 0.294;* F*(2, 6) = 1.109, *p* = 0.389, respectively (One-way ANOVA). *N* = 3 mice for each group. **B** Immunoblot of proteins from mice at PD230 involved in cone phototransduction shows comparable protein expression in WT and homozygous *Arr3*^*P2ACreERT2*^ mice, quantified by ARR3, M-opsin, and S-opsin from left to right, *p* = 0.919, 0.764, 0.688, respectively. *N* = 3 mice for each group. **C** qPCR analysis of gene expression for *Arr3, Cnga3, Opn1mw* in WT and homozygous at PD55 (left), *p* = 0.057, 0.057, 0.857, respectively; and PD230 (right), *p* = 0.700, 0.400, 0.400, respectively*. N* = 4 for WT and homozygous group at PD55. *N* = 3 for WT and homozygous groups at PD230. Black dots indicate WT, green dots indicate heterozygous, and red dots indicate homozygous *Arr3*^*P2ACreERT2*^ mice. Data represent mean ± 2SE in **A**, **B**, and **C**. *: *p* < 0.05; **: *p* < 0.01; ***: *p* < 0.001

To test whether the KI *P2A-CreER*^*T2*^ cassette in *Arr3* affects ARR3 protein expression, which would cause cones to be unlabeled by the anti-ARR3 antibody in IHC as we observed above, we first examined the cone-specific protein expression by performing immunoblot analysis of retina lysates obtained from WT, heterozygous, and female homozygous mice at PD55. We then probed the blots with the anti-ARR3 antibody. There was no significant difference, *F*_2, 6_ = 0.756, *p* = 0.510, (One-way ANOVA) (Fig. 6A). (*N* = 3 mice in each group). To further test whether the KI P2A-CreER^T2^ cassette could affect other cone-specific proteins that interact with ARR3, we performed immunoblotting with anti-M-opsin and anti-S-opsin antibodies, which also showed no significant differences amongst mice of different genotypes, *F*_2, 6_ = 1.514, *p* = 0.294; *F*_2, 6_ = 1.109 *p* = 0.389, respectively (One-way ANOVA) (Fig. 6A). (*N* = 3). To further examine whether there was a slow reduction in the expression of cone-specific proteins with age, immunoblot analysis was performed on retinal lysates from mice at PD230. Again, no significant differences in ARR3, M-opsin and S-opsin expression between WT and homozygous mice, *p* = 0.919, 0.764, and 0.688, respectively (Fig. 6B) were observed. Like in the *Arr3*^*T2ACreERT2*^ mouse, the anti-ARR3 antibody could recognize the denatured P2A modified ARR3 protein in immunoblot analysis when it was denatured, however, the same anti-ARR3 antibody could not recognize the ARR3 protein modified by P2A at the C-terminus in IHC staining. This phenomenon is consistent in *Arr3*^*T2ACreERT2*^ and *Arr3*^*P2ACreERT2*^ mice. To test whether *Arr3* mRNA expression is unaffected by the KI cassette, we quantified *Arr3* mRNA expression by quantitative real-time RT-PCR (qPCR). Examination of *Arr3* expression showed a significant reduction in *Arr3* mRNA levels in homozygous mice as compared with WT mice at PD55 (*N* = 4 for WT and 4 for homozygous mice, *p* = 0.001) and PD230 (*N* = 3 for WT and homozygous mice, *p* = 0.03) (Fig. 6C). However there was no progressive reduction in *Arr3* level with age. Further qPCR analysis of *Cnga3 and Opn1mw* at PD55 and PD230 revealed that mRNA levels were comparable between genotypes (Fig. 6C). Together, the results from immunoblots and qPCR demonstrated that the KI P2A-CreER^T2^ cassette affects *Arr3* mRNA at PD55 and PD230. However, there is no significant difference in ARR3, M-opsin, S-opsin expression, or *Cnga3 and Opn1mw* mRNA levels at PD55 and PD230.

### *Impact of the KI P2A-CreER*^*T2*^*cassette on retinal structure inArr3*^*P2ACreERT2*^*mice*

**Fig. 7:**
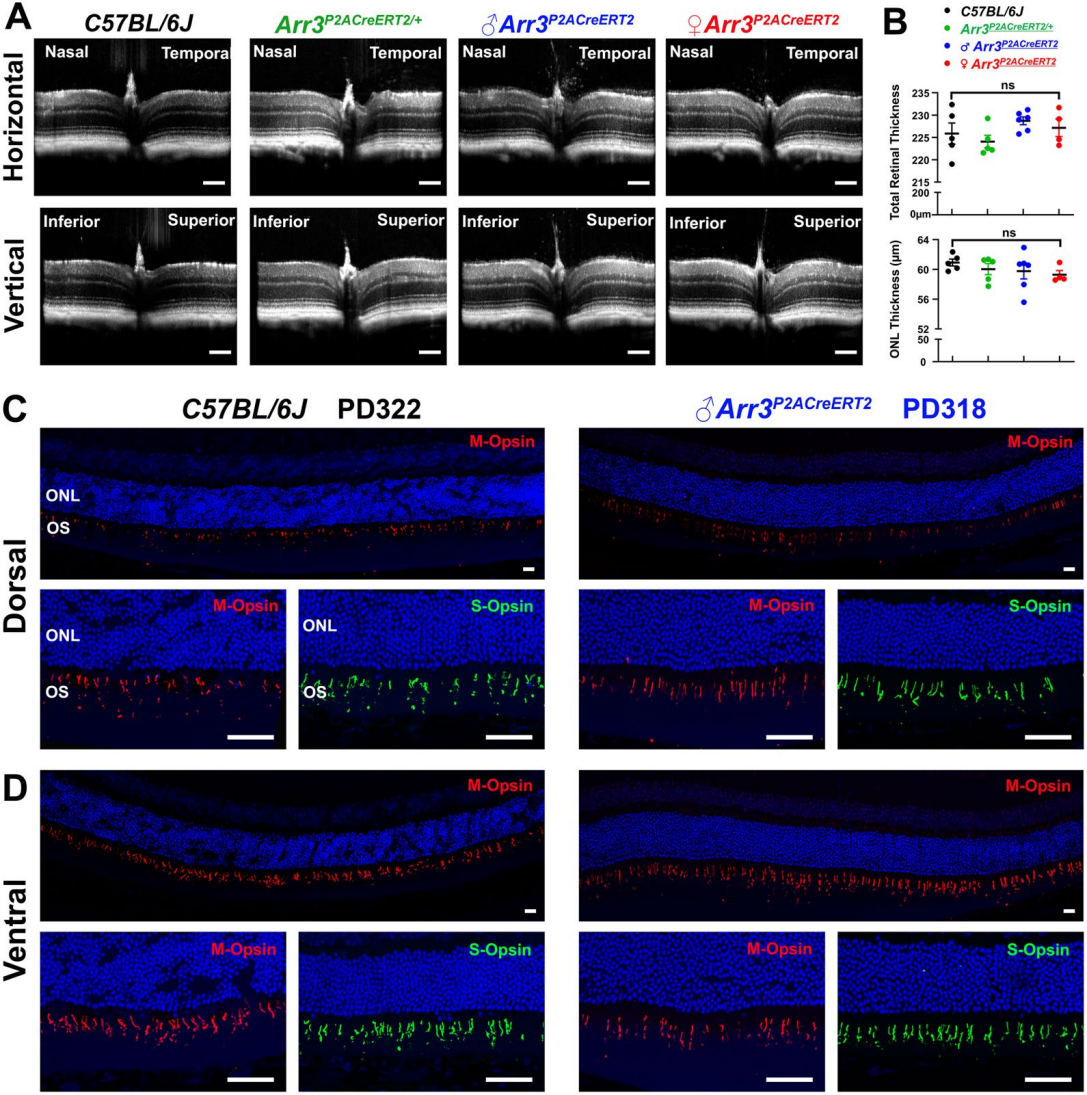
Intact retina/cone structure in *Arr3*^*P2ACreERT2*^ driver. **A** Horizontal and vertical retinal OCT images from representative 2 month-old WT, female heterozygous *Arr3*^*P2ACreERT2/*+^, male hemizygous and female homozygous *Arr3*^*P2ACreERT2*^ mice. **B** Quantification of the total retinal and outer nuclear layer thickness showed no significant difference between the four groups (One-way ANOVA, Additional file 1: Table S4). Data represent mean ± 2SE. *N* = 5, 5, 6, and 4 mice for WT, female heterozygous, male hemizygous, and female homozygous *Arr3*^*P2ACreERT2*^ mice, respectively. The mean total retinal thicknesses were 225.86, 224.05, 228.70, and 227.15 μm, respectively. The mean outer nuclear layer thicknesses were 60.92, 60.03, 59.77, and 59.28 μm, respectively. **C**–**D** Representative retinal sections from a PD322 WT and a PD318 male hemizygous *Arr3*^*P2ACreERT2*^ mouse were stained with cone markers (antibodies against M-opsin and S-opsin). Fewer M-opsin positive (red) and S-opsin positive (green) cones were present in the dorsal retinal compared to cones in the ventral retinal in both WT and *Arr3*^*P2ACreERT2*^ mice. *ONL* outer nuclear layer, *OS* outer segment, *ns* not statistically significant. Scale bars, 200 μm in **A**, and 20 μm in **C** and **D**

Examination of retinal histology at PD364 revealed a comparable thickness of the total retinal and outer nuclear layer in WT and *Arr3*^*P2ACreERT2*^ mice, demonstrating that retinal structure was unaffected by the KI P2A-CreER^T2^ cassette. To support this observation, we further analyzed the retinal structure using spectral domain optical coherent tomography (SD-OCT) in WT, female heterozygous *Arr3*^*P2ACreERT2/*+^, male hemizygous, and female homozygous *Arr3*^*P2ACreERT2*^ mice at two-months-old (Fig. 7A). The total mean retinal thicknesses were 225.86, 224.05, 228.70, and 227.15 μm, respectively and the mean outer nuclear layer thicknesses were 60.92, 60.03, 59.77, and 59.28 μm, respectively. A one-way ANOVA did not reveal a significant difference on either total retinal (*F*_3,16_ = 1.515, *p* = 0.249) or outer nuclear layer (*F*_3,16_ = 0.668, *p* = 0.584) thickness (Fig. 7B).* N* = 5, 5, 6, and 4 mice for WT, female heterozygous, male hemizygous, and female homozygous *Arr3*^*P2ACreERT2*^ mice, respectively. Because cone cells contribute 3–5% of photoreceptors in rod dominant retina in mice, the subtle cone cell loss may not be observed in the outer nuclear layer thickness or total retinal thickness. To examine the density of cone cells, IHC studies were done on retinal sections stained for M-opsin and S-opsin on dorsal and ventral retinae from male hemizygous *Arr3*^*P2ACreERT2*^ mouse at PD318, which showed comparable densities of M-opsin positive (red) and S-opsin positive (green) cones in the same regions at defined distances from the optic nerve with WT mouse at PD322 (Fig. 7C, D). Together, these results support the conclusion that the KI P2A-CreER^T2^ cassette in *Arr3*^*P2ACreERT2*^ mice does not affect the retinal structure or cone density.

### *Impact of the KI P2A-CreER*^*T2*^* cassette on retinal function in Arr3*^*P2ACreERT2*^* mice*

Subsequently, we determined whether the KI P2A-CreER^T2^ cassette at the C-terminus of the ARR3 protein affects cone function. By following the same ERG protocol, we tested scotopic and photopic responses on the WT, heterozygous, and homozygous *Arr3*^*P2ACreERT2*^ mice at two months old. No significant difference in scotopic and photopic *a-* and *b-*wave amplitudes was recorded (Additional file 1: Fig. S1). To further assess the major ON- and OFF-bipolar cell pathways, we designed an extensive ERG protocol, which includes additional 12 steps of flicker ERG frequency series before light adaptation (LA).

**Fig. 8:**
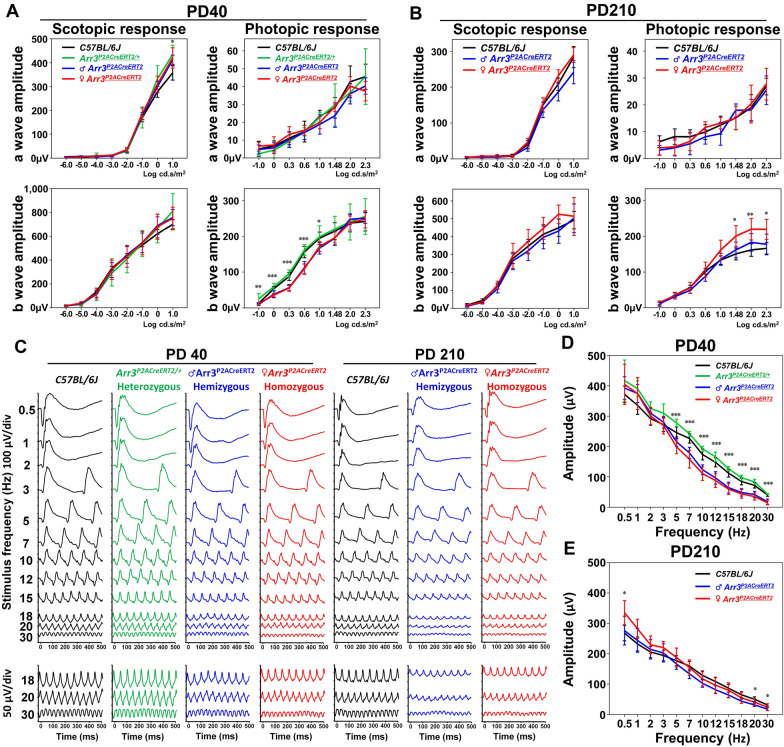
Intact retinal function in *Arr3*^*P2ACreERT2*^ driver. **A** At PD40, mice with KI alleles had larger scotopic *a-* wave amplitudes only at 1.0 log cd.s/m^2^ intensity; *F*(3,21) = 3.414, *p* = 0.036 (One-way ANOVA, Additional file 1: Table S5) (*A, left upper*). There was no significant difference in scotopic b- wave and photopic *a-* wave amplitudes between mice of four different genotypes (*A, left lower, right upper*). Male hemizygous and female homozygous mice had smaller photopic *b*- wave amplitudes compared to the *b-* wave amplitudes of heterozygous and wild-type mice at −1.0, 0, 0.3, 0.6, and 1.0 log cd.s/m^2^ intensities (One-way ANOVA, Additional file 1: Table S5) (*A, right lower*). **B** At PD210, female homozygous mice had large photopic b- wave amplitudes at 1.48, 2.0 and 2.3 log cd.s/m^2^ intensities (One-way ANOVA, Additional file 1 Table S6). There was no difference in scotopic *a-*, *b-*, and photopic *a-* wave amplitudes in mice of three different genotypes (*B, upper*). (**C**) Representative flicker series recordings from mice of different genotypes at PD40 an PD210. (**D**–**E**) Male hemizygous and female homozygous mice tended to have larger amplitudes at low stimulus frequencies and lower amplitudes at higher stimulus frequencies than wild-type mice. These differences are significant in younger mice (PD40) at 5, 7, 10, 12, 15, 18, 20 and 30 Hz frequencies (*D*) and in older mice (P210) at 0.5, 20 and 30 Hz frequencies (*E*) (One-way ANOVA, Additional Table S7). Data represent mean ± 2SE. For PD40, *N* = 7, 3, 7, and 8 mice for WT, female heterozygous *Arr3*^*P2ACreERT2/*+^, male hemizygous and female homozygous *Arr3*^*P2ACreERT2*^ mice, respectively. For PD210, *N* = 11, 6, and 6 mice for *C57BL/6 J*, male hemizygous and female homozygous *Arr3*^*P2ACreERT2*^ mice, respectively. *: *p* < 0.05; **: *p* < 0.01; ***: *p* < 0.001

At PD40, mice with KI alleles had larger scotopic *a-* wave amplitude than WT at 1.0 log cd.s/m^2^ intensity; *F*_3,21_ = 3.414, *p* = 0.036 (One-way ANOVA) (Fig. 8A, left upper). However, photopic *b-*wave amplitudes from male hemizygous and female homozygous mice were smaller than heterozygous and WT mice at intensities no higher than 1.0 log cd.s/m^2^. These results differed from the results at PD60 by using the protocol without additional 12 steps of flicker series before the LA (Fig. 8A, right lower; Fig. S1, right lower). The inconsistent findings of photopic *b-* wave amplitudes between PD40 and PD60 could be attributed to the additional 12-step flicker series and/or ages of mice.

It has been demonstrated that in mice with cone arrestin knockout (*Arr4*^*−/−*^), the photopic *a-* and *b-* waves’ amplitudes in old mice (defined as 7 months old) were significantly decreased compared to age-matched WT mice [32]. To examine whether cone degeneration is present in our *Arr3*^*P2ACreERT2*^ mice at the same age, we tested our mice at PD210 using the extended protocol previously tested on young mice. Although homozygous *Arr3*^*P2ACreERT2*^ mice had large amplitudes in scotopic *a-*, *b-* and photopic *b-*waves, the difference was significant at intensities of 1.48, 2.0, and 2.3 log cd.s/m^2^ (*F*_2,20_ = 5.574, *p* = 0.012; *F*_2,20_ = 6.587, *p* = 0.06; and *F*_2,20_ = 5.867, *p* = 0.01, respectively) (Fig. 8B, right). It is noteworthy that in our homozygous *Arr3*^*P2ACreERT2*^ mice at PD210, scotopic and photopic serial intensities ERG didn’t reveal significant reduction in *a-* or *b-* wave amplitudes, on the contrary, the photopic *b-* wave amplitudes were significantly increased at intensities of 1.48, 2.0, and 2.3 log cd.s/m^2^. Moreover, we tested extended ERG in one male hemizygous mouse at PD300, which was the oldest mouse at the time when we were preparing this manuscript. The amplitudes of scotopic serial intensities, flicker series, and photopic serial intensities were indistinguishable compared to the amplitudes of WT mice at the same age (Additional file 1: Fig. S2).

In general, the response at 0.5 Hz was similar to the scotopic ERG at the same luminance in terms of the existence of components that correspond to the *a-* and *b-* waves of the single flash ERG (Fig. 8C). At low stimulus frequencies, such as 0.5, 1 and 2 Hz, male hemizygous and female homozygous mice had non-significantly larger amplitudes than WT mice, however, above 6 Hz, male hemizygous and female homozygous mice had smaller amplitudes than WT mice, which are more significant in mice at PD40 (Fig. 8 D, E). Taken together, results from scotopic, photopic serial intensity ERG, and flicker ERG frequencies series up to ten-month-old demonstrate that *Arr3*^*P2ACreERT2*^ mice do not exhibit signs of cone or photoreceptor degeneration caused by the KI P2A-CreER^T2^ cassette at the C-terminus of ARR3. However, reduced amplitudes at stimulus frequencies above 6 Hz indicates that cone bipolar pathways were affected, which results in smaller photopic *b-* wave amplitudes at intensities no higher than 1.0 log cd.s/m^2^ at PD40.

## Discussion

In this study, we used a BAC recombineering method to knock in the “CreER^T2^” sequence into the *Gnat2* and *Arr3* genes, and generated three novel inducible CreER^T2^ mice with high cone cell specificity. The *Gnat2*^*CreERT2*^ mouse we generated has two limitations for future application: first there are low levels of Cre-loxP recombination in the heterozygous state and second, there is complete cone dysfunction when it is bred into the homozygous state. Our group generated a valuable rod-specific Cre line, the *Pde6g*^*CreERT2*^ mouse, using the same strategy [34], which showed nearly complete Cre-loxP recombination from the native *Pde6g* allele and normal retinal ERG in the heterozygous state [34]. However, the *Gnat2*^*CreERT2*^ mouse showed low-level Cre-loxP recombination in the heterozygous state, which greatly diminishes its use as a tool for future research even though the ERG responses were indistinguishable from litter control WT mouse. The genetic engineering design, which inserts a “CreER^T2^” sequence into the translational start codon, pushes the following coding sequence further away from its original promoter and regulatory elements and impairs gene function from that KI allele. This likely caused complete cone and rod dysfunction in homozygous *Gnat2*^*CreERT2*^ and *Pde6g*^*CreERT2*^ mice respectively.

*Arrestin 3* is highly expressed in the mammalian cone photoreceptors [35]. Mouse cones have Arr3 and Arrestin 1 (ARR1) [36]. Although ARR3 accounts for only 2% of arrestins in mouse cone photoreceptors, it is not expressed in mouse rods. Furthermore, it has a stronger affinity for cone opsins compared to ARR1 and plays an important role in terminating photoactivated signals originating from cone opsins [35, 37–40]. Previous studies using RNA in situ hybridization have demonstrated that ARR3 and PNA, the only two cone markers, were detected as early as embryonic day (E) 15.5 [41]. In addition, ARR3 detection persisted through age PD60 [41]. Recently, the *Arr3* gene has been used as cone-specific marker to determine clustering and marker identification in single-cell RNA-sequencing. Therefore, *Arr3* is specifically and consistently expressed in cones [42]. In our *Arr3*^*T2ACreERT2*^ and *Arr3*^*P2ACreERT2*^ mice, we found Cre-loxP recombination could be successfully induced by delivering tamoxifen either through intraperitoneal injections or intragastric injections in neonatal mice (PD2 ~ PD5). *Arr3*^*P2ACreERT2*^ mice may be particularly useful in studying cone specific function and physiology at earlier stages. Use of this mouse line during embryonic stages will require additional validation studies.

Mary Lyon first proposed X-chromosome inactivation (also known as lyonization), which is a dosage-compensating effect of genes located on the X chromosome in XX individuals [29, 43]. X chromosome inactivation in XX mammals involves different expression of two homologous chromosomes within the same nucleoplasm. This process occurs through transcriptional inactivation of one of the X chromosomes and results in the shutdown of that allele [29, 44]. Because the *Arr3* gene is located on X-chromosome, we observed mosaicism in SW-AF images acquired from *Arr3*^*P2ACreERT2/+*^*Ai14D*^*+/-*^ mice, which in SW-AF images phenocopies the mosaic retinopathy in carriers of hereditary X-linked recessive diseases [45]. All the cells in female *Arr3*^*P2ACreERT2/+*^*Ai14D*^*+/-*^ mice have one *Arr3*^*P2ACreERT2*^ KI X-chromosome and one native *Arr3* X-chromosome. According to the theory of XCI [29], only one X-chromosome is activated while the other is inactivated due to long non-coding RNA *Xist *[46, 47]. This “mosaic” SW-AF phenomenon in female heterozygous mice could be explained by the XCI while the mosaic-like pattern observed in SW-AF images in male hemizygous *Arr3*^*T2ACreERT2*^ mice is likely caused by inefficient Cre-LoxP recombination. In our study, it was planned to determine whether administration of tamoxifen would induce Cre-loxP recombination and a mosaic pattern of SW-AF in female heterozygous mice; however, it caught our attention in IHC staining that tdTomato-positive cone cells could not be labelled by anti-ARR3 antibody, perhaps because the antigen was modified by 2A peptides.

The 2A peptides, 18–22 amino acids on average, have been used widely in place of the internal ribosomal entry site (IRES). These peptides overcome the disadvantages of IRES elements, such as their long length (~ 500 nucleotides), the inability of connecting more than one protein, and lower translation efficiency of genes placed after the IRES compared to genes located before IRES. These 2A peptides commence self-mediated cleavage (ribosomal skipping) during the translation by breaking the peptide bond between the Glycine and Proline at their C-terminus [48, 49]. As a result, the upstream protein will have a few extra 2A residues added to its C terminus while the downstream protein will have an extra proline added to its N-terminus. In the two *Arr3*^*2ACreERT2*^ mice, we found that the anti-ARR3 antibody targets an epitope within 12 amino acids of ARR3 protein from the C-terminus. Immunoblotting with anti-ARR3 can identify the denatured ARR3 protein that has been modified by 2A peptides, whereas it failed to label the same modified ARR3 protein when it retained its tertiary structure. It remains possible that the C-terminal modification by these 2A peptides altered the tertiary structures of ARR3 proteins, causing the cones to be unlabeled by the anti-ARR3 antibody in IHC. Although the ARR3 protein expression in immunoblot staining was not affected by the KI 2A-CreER^T2^ cassette in our two *Arr3*^*2ACreERT2*^ mice, a similar design with KI P2A-CreER^T2^ in *Rpe65*^*P2ACreERT2*^ mice demonstrated reduced RPE65 protein expression in immunoblotting analysis [50]. The mice generated using this design, including the *Rpe65*^*P2ACreERT2*^ and *Arr3*^*P2ACreERT2*^ mice, showed a reduction in mRNA levels even though there were no signs of structural or functional retinal pathology in *Rpe65*^*P2ACreERT2*^ [50] and *Arr3*^*P2ACreERT2*^ mice. Therefore, the effects of the KI cassette using these 2A peptides on protein expression and mRNA levels vary with different upstream or downstream genes.

Visual arrestins are required to close the G-protein-coupled receptor (GPCR)-light-activated phototransduction cascade in photoreceptors. One of the major roles of ARR3 protein is to shutoff the cone phototransduction cascade through the binding of the C-terminus of ARR3 with phosphorylated red/green opsins. The cone arrestin knockout (*Arr4*^*−/−*^) mouse is an example of a mouse model of cone degeneration due to loss of cone arrestin proteins. Increased amplitudes of photopic *a-* waves at 2 months old occurred because of the inability to terminate cone phototransduction. Reduced photopic *a-*wave amplitudes at 7 months old were likely due to cone cell loss [32]. Amplitudes of photopic a-waves are usually used as indicators of cone function because the a-wave originates in the photoreceptor layer [51]. In our *Arr3*^*P2ACreERT2*^ mice, the KI P2A-CreER^T2^ to the C-terminus of the ARR3 protein could potentially affect cone function. However, we found no significant increase in the amplitudes of photopic *a-*waves at PD40; nor were amplitudes of photopic *a-* waves reduced in *Arr3*^*P2ACreERT2*^ mice compared to the amplitudes of age-matched WT mice as old as 10 months (Fig. 8 and Additional file 1: S2). Together, the results from immunoblotting and IHC staining (Figs. 6, 7) support our conclusion that there is no detectable cone degeneration or loss in our *Arr3*^*P2ACreERT2*^ mice.

We found the photopic *b-* wave amplitudes from male hemizygous and female homozygous mice at PD40 were smaller than heterozygous and WT mice at intensities that were not higher than 1.0 log cd.s/m^2^ (Fig. 8A, right lower). These results differ from those at PD60 using the protocol without additional 12 steps of flicker series before the LA (Additional file 1: Fig. S1, right lower). Because the ERG *b-*wave originates in retinal cells that are post-synaptic to the photoreceptors [51], reduced photopic *b-* wave amplitudes indicate that the bipolar cell pathways were likely affected by the additional flicker series before the LA which resulted in noisy signals from activated opsins. The 12-step flicker series used in this study contains three frequency ranges that are dominated by rod bipolar pathways (below 5 Hz), cone ON- bipolar pathway (between 5 and 15 Hz) and cone OFF- bipolar pathway (above 15 Hz) [52]. It is worth noting that the hemizygous and homozygous mice had smaller flicker ERG amplitudes than WT mice at frequencies above 6 Hz at PD40 and PD210 (Fig. 8D, E), indicating that only the cone-driven ON- and OFF- bipolar pathways were affected. Comparing the flicker series ERG in young (PD40) and old age (PD210) mice, cone bipolar pathways were more affected in young mice than old mice, which subsequently is shown in the reduced photopic *b*- wave amplitudes at intensities no more than 1.0 Log cd.s/m^2^ after LA at PD40 (Fig. 8A, right lower). Comparing the photopic *b*- wave amplitudes in mice with and without this “additional 12-step flicker ERG” at PD40 (Fig. 8A) and PD60 (Additional file 1: Fig. S1) respectively, photopic *b-* wave amplitudes in younger mice were more likely to be affected by this “additional 12-step flicker ERG”. From the results in our study, the KI P2A-CreER^T2^ to the C-terminus of ARR3 protein did not affect the cone cell function (photopic *a-* wave amplitudes) directly, however, the cone-driven ON- and OFF- bipolar pathways were affected as we observed the reduced amplitudes in flicker ERG (Fig. 8D, E). Moreover, photopic *b*- wave amplitudes (postsynaptic responses) in younger mice (PD40) were more susceptible to this “additional 12-step flicker ERG” than in older mice (PD210). Without this additional flicker ERG before the LA, there is no significant difference in photopic *b*- wave amplitudes between three different genotypes.

In summary, we report the generation of three novel cone-specific CreER^T2^ mice by knocking in the “CreER^T2^” sequence into *Gnat2* and *Arr3* genes respectively with the BAC recombineering. The *Gnat2*^*CreERT2*^ mouse was generated by introducing the “CreER^T2^” sequence into the translational START codon of the *Gnat2* gene, which revealed a 10 to 15% Cre-LoxP recombination rate in heterozygous state after tamoxifen induction and extinguished cone responses in the homozygous state*.* The *Arr3*^*T2ACreERT2*^ mouse was engineered by introducing the “T2A-CreER^T2^” sequence into the translational STOP codon of the *Arr3* gene. Although the KI T2A-CreER^T2^ cassette does not affect the retinal/cone function or cone protein expression, the cone cells in this mouse line could not be labeled by anti-ARR3 antibody in IHC staining, which could be attributed to the modification of the 2A peptides at the C-terminus of ARR3 protein. However, the Cre-LoxP recombination efficiency was 40% in the hemizygous state after tamoxifen induction. The *Arr3*^*P2ACreERT2*^ mouse was engineered using the same method as the *Arr3*^*T2ACreERT2*^ mouse by changing the “T2A-CreER^T2^” sequence to “P2A-CreER^T2^”. Cre-LoxP recombination was highly tamoxifen dependent and could be achieved with either intragastric injection in neonatal mice or intraperitoneal injection in adult mice. Although the *Arr3* mRNA expression was lower than we anticipated, immunoblot analysis revealed comparable ARR3 protein expression. We did not detect any pathologic effects in retina/cone structure and function resulting from the KI P2A-CreER^T2^ cassette in this mouse line up to 10 months of age. Because cones make up 3–5% of the photoreceptors in human and mouse retina, future research will benefit from this *Arr3*^*P2ACreERT2*^ mouse line, allowing for the ability to determine the cellular metabolism between cones, rods, and retinal pigment epithelium in normal physiology, and degenerative retinal disorders, such as retinitis pigmentosa.

## Conclusions

We expect the *Arr3*^*P2ACreERT2*^ mouse to be a valuable line in studying cone cell biology, function, as well as its relationship with rod and other retinal cells. Moreover, the Cre activity can be induced by delivering tamoxifen intragastrically as early as PD2, which will be useful for studying retinal development or in rapid degenerative mouse models. Considering that the *Arr3* gene is located on the X-chromosome and 50% of the cone cells may express Cre recombinase in female heterozygous mice, experiments using male hemizygous or female homozygous mice will likely achieve nearly complete Cre-LoxP recombination in the cone photoreceptors.

## Methods

### Mouse care and housing

All the mice used in this study were housed under a 12 h light/ 12 h dark cycle in the animal facility (Institute of Comparative Medicine) and handled by complying with the Statement for the Use of Animals in Ophthalmic and Vision Research of the Association for Research in Vision and Ophthalmology. All the experiments were approved by the Institutional Animal Care and Use Committee at Columbia University Irving Medical Center. C57BL/6 J (JAX 000664, *B6/J or* WT), B6.Cg-Tg(ACTFLPe)9205Dym/J (JAX 005703, *ACTB*-*FLPe*) and B6.Cg-*Gt(ROSA)26Sor*^*tm14(CAG−tdTomato)Hze*^/J mice (JAX 007914, *Ai14*) were purchased from the Jackson Laboratory (Bar Harbor, ME, USA). All the mice in this study were on the *B6/J* background and had been genotyped to exclude the contamination of *Crb1*^*rd8*^ and *Pde6β*^*rd1*^ [53, 54].

### Generation of ***Gnat2 ***^*CreERT2*^ mice using embryonic stem (ES) cell gene targeting and genotype

A CreER^T2^ with an FRT-flanked neomycin cassette (hereafter *“CreER*^*T2*^*-FNF”*) replacing the translation START codon (ATG) of *Gnat2* gene was engineered using bacterial artificial chromosome (BAC) recombineering (Fig. 1A). A gene targeting vector was constructed by retrieving 2 kb upstream of and 5 kb downstream of the *CreER*^*T2*^*-FNF* cassette insertion. The gene-targeted vector was linearized with Ascl and electroporated into KV1 (129B6N hybrid) ES cells. After G418 selection, one of the six identified targeted ES cell clones was injected into C57BL/6 J blastocysts to generate chimeric mice. Male chimeras were bred to *ACTB*-*FLPe* females for germline transmission and neomycin cassette removal. Mice were genotyped and verified using polymerase chain reaction (PCR) analysis, using the primer set targeting the junction of the knock-in fragment (Additional file 1: Table S1). The PCR products were resolved via 0.8% agarose gel electrophoresis using GelGreen^™^ (Biotium) as the visualizing dye. The DNA bands were visualized using an iBright^™^ FL1500 Imaging System (Invitrogen).

### Generation of *Arr3*^*2ACreERT2*^ mice using embryonic stem (ES) cell gene targeting and Genotype

A 2A peptidie-CreER^T2^ and an FRT-flanked neomycin cassette (hereafter *“2A-CreER*^*T2*^*-FNF”*) were introduced into translation STOP codon (TAG) using bacterial artificial chromosome (BAC) recombineering (Fig. 2A). A gene targeting vector was constructed and electroporated into KV1 (129B6N hybrid) ES cells as described above. Genotyping was performed as above using the primer set targeting the wild-type allele and the junction of the knock-in fragment (Additional file 1: Table S1).

### Tamoxifen induction

The mice were intraperitoneally injected with tamoxifen (T5648; Signa-Aldrich) at 100 μg/g for three consecutive days. Intragastric delivery was applied to neonatal mouse pups with a 27-gauge needle through the abdominal wall for three consecutive days.

### In vivo Electroretinography (ERG)

ERG recordings were obtained using an Espion V6 Diagnosys ColorDome (Diagnosys LLC; Lowell, MA, USA) with simultaneously both eye stimulation and voltage recording. All mice were dark adapted (> 12 h) before the ERG procedure and all steps were performed under dim red illumination as described previously [55, 56]. Mice were anesthetized just before analysis by a mixture of ketamine (100 mg/Kg) and xylazine (10 mg/Kg). Tropicamide (1%) and phenylephrine hydrochloride (2.5%) were used to dilate the pupils, and Gonak-Hypromellose Ophthalmic Solution (2.5%) was used to keep the eyes hydrated. The scotopic single flash serial intensity ERG consisted of eight steps using a white flash ranging in intensity from − 6.0, − 5.0, − 4.0, − 3.0, − 2.0, − 1.0, 0.0, and 1.0 log cd.s/m^2^ in sequence. For intensities from − 6.0 to − 4.0 log cd.s/m^2^, responses were averaged from twenty sweeps collected at intervals of 2 s, for − 3, − 2, − 1 and 0 log cd.s/m^2^, 10 sweeps were collected at intervals of 10 s, for 1 log cd.s/m^2^, three sweeps were collected at intervals of 20 s. The photopic single flash serial intensity ERG consisted of a 10-min adaptation step to the 30 cd/m^2^ white light background followed by eight steps using a white flash ranging in intensity from − 1.0, 0.0, 0.3, 0.6, 1.0, 1.48, 2.0, and 2.3 log cd.s/m^2^ in the sequence above this background. For intensities from −1.0 and 0.0 log cd.s/m^2^, responses were averaged from 10 sweeps collected at intervals of 1 s, for 0.3, 0.6, 1.0, 1.48 log cd.s/m^2^, 10 sweeps were collected at intervals of 5 s, for 2.0 and 2.3 log cd.s/m^2^, five sweeps were collected at intervals of 5 s.

To assess bipolar cell pathways together with photoreceptor function, we designed an extended ERG protocol that could record responses stimulated by scotopic serial intensities, flicker series, and photopic serial intensities. Different from the ERG protocols used for *Gnat2*^*CreERT2*^ and *Arr3*^*T2ACreERT2*^ mice, this extensive ERG protocol includes additional 12 steps of flicker series, which can assess the major ON- and OFF- bipolar cell pathways. The flash flicker series consisted of 12 steps using 0.5 log cd.s/m^2^ flickering white light with varying frequency (0.5, 1, 2, 3, 5, 7, 10, 12, 15, 18, 20 and 30 Hz) without any background illumination (0 cd/m^2^) [52], for frequencies from 0.5 to 3 Hz, response, 20 sweeps were collected, for 5–15 Hz, 30 sweeps were collected, for 18–20 Hz, 40 sweeps were collected, and for 30 Hz, 50 sweeps were collected. There was no delay between sweeps. Band-pass filter frequencies were 0.3 and 300 Hz for single-flash serial intensity and flicker ERG at all frequencies. The a-wave amplitudes were measured from the baseline to the trough of the *a*-wave, and the *b*-wave amplitude was measured from the trough of the *a*-wave to the peak of the *b*-wave. Flicker response amplitudes were measured from the trough to the peak of each response at all frequencies. The responses recorded simultaneously from both eyes were averaged as data to represent each mouse for further analysis. All the “*N*” in this manuscript represents the number of mice.

### Spectral domain optical coherence tomography (SD-OCT) image acquisition

In vivo retinal images were acquired using an Envisu UHR2200 (Bioptigen, Durham, NC) with theoretical axial resolution in the tissue of 1.75 µm. Mice were anesthetized, eyedrops were used to dilate the pupils and keep the eyes hydrated as described above. Mice were positioned to have the eye look directly into the lens, and then to align the optic nerve head to be on the center of the OCT scan horizontally and vertically. A rectangular scan with a 1.8 mm length and width, 0° angle, 0 mm horizontal and vertical offsets 1000 lines of A-scans/B-scans, 100 B-scans, 10 frames/B-scan, 80 lines of inactive A-scans/B-scan, and 1 volume was captured. Ten-frame OCT images were averaged using Bioptigen InVivoVue^®^ and the averaged image was subjected to auto-segmentation for retinal layer thickness measurement using Bioptigen Diver^®^ V. 3.4.4 software.

### Histology and immunohistochemistry (IHC) staining

Eyes were enucleated from euthanized mice, followed by cautery on the limbus to create a mark at the 12 o’clock position and 4% paraformaldehyde fixation. The hematoxylin and eosin stain (H and E) and IHC staining on retinal whole mounts and cryosections were carried out as previously described [55, 57, 58].

For IHC analysis of cone cells, anti–ARR3 (1:1000, AB15282, Sigma-Aldrich), anti-PNA (1:200, FL-1071, Vectorlabs), anti–Cre Recombinase (1:200, #15,036, Cell Signaling), anti–GlyPh (1:500; kind gift from Dr Pfeiffer-Guglielmi, University of Tübingen, Germany) [31], anti–M-opsin (1:200, AB5105, Sigma-Aldrich), and anti–S-opsin (1:200, AB5407, Sigma-Aldrich) antibodies were used. The secondary antibody used was Cy^™^2 AffiniPure Donkey anti-rabbit antibody (dilution, 1:200; Jackson ImmunoResearch). Nuclei were labeled with Hoechst 33,342 (62,249; Thermo Scientific). After being washed three times with PBS, the sections were mounted with fluorescence mounting medium (S3023; Agilent) and viewed using a Nikon confocal microscope (A1RMP, Nikon Corporation, Tokyo, Japan). Image acquisition and processing for three-dimensional rendering was performed using NIS-Elements ver. 4.1 (Nikon).

### Immunoblotting analysis

Immunoblot analysis was performed as described previously [55]. Retinas were homogenized in RIPA lysis and extraction buffer (89,900; Thermo Scientific) containing protease inhibitor cocktail (P8340; Sigma-Aldrich). The lysate was collected after sonication and centrifugation at 1000 ×*g* and 4 °C for 10 min. 20 µg of protein was mixed with 4 × Laemmli Sample buffer (1610747; Bio-Rad) with β-mercaptoethanol (M3148; Millipore Sigma), resolved by SDS–polyacrylamide gelelectrophoresis using 4–15% BIO-RAD TGX pre-cast gels (4561083; Bio-Rad), then transferred to nitrocellulose membranes (Bio-Rad). After blocking the membrane with EveryBlot blocking buffer (12010020; Bio-Rad), the primary antibodies (anti–ARR3, 1:2000, AB15282, Sigma-Aldrich; anti–M-opsin, 1:1000, AB5105, Sigma-Aldrich; anti–S-opsin, 1:1000, AB5407, Sigma-Aldrich; anti-β-actin 1:2500, 8H10D10, Cell Signaling Technology) were applied for overnight at 4 °C. After rinsing in 0.1% PBST, the nitrocellulose membranes were incubated with mouse monoclonal anti-rabbit IgG-HRP secondary antibody (1:3000; ab99697; Abcam) or rabbit anti-mouse IgG-HRP secondary antibody (1:3000, ab6725; Abcam) for 1 h at room temperature. Protein expression was visualized by enhanced chemiluminescence using Immobilon Western (WBKLS0100; EMD Millipore) and the iBright Imaging System (FL1500; Invitrogen). Signal intensities were quantified by densitometric analyses using iBright Analysis Software (version 4.0.1; Invitrogen).

### Quantitative real-time RT-PCR (qRT-PCR)

Total RNA was extracted from freshly dissected mouse retinas using the RNeasy Mini Kit (74,104; QIAGEN). Reverse transcription to cDNA and real-time qPCR amplification was performed by following the protocols as previously described [55]. The primer sets were listed in Additional file 1: Table S2. *Gapdh* was used as the housekeeping gene.

### Statistical analysis

All the data were analyzed in a double-blind fashion. Statistical significance was determined by unpaired student *t*-test and one-way ANOVA. *p* < 0.05 was considered statistically significant. Data visualizations were performed by the SPSS (IBM SPSS Statistics for Windows, Version 27.0, IBM Corp. Armonk, NY) and GraphPad Prism 9. Statistical data is listed in the Additional datasets (Additional file materials).

### Study approval

The study was conducted according to previously established IACUC guidelines and approved by the Institutional Animal Care and Use Committee of Columbia University (IACUC: AABE6580).

## Supplementary Information

**Additional file 1: Figure S1.** Scotopic and photopic serial intensity ERG responses from 2 month-old *C57BL/6J*, *Arr3*^*P2ACreERT2/+*^, and* Arr3*^*P2ACreERT2*^ mice. **Figure S2.** Representative Scotopic, photopic serial intensity ERG and flicker ERG frequency series responses from 10 month-old C57BL/6J and Arr3^P2ACreERT2^ mice. **Figure S3.** Cre-LoxP recombination in* Gnat2*^*CreERT2/+*^*Ai14D*^*+/-*^ mice. **Figure S4.** Delayed induction (at 5 months old) of cone-specific Cre-LoxP recombination activity in *Arr3*^*P2ACreERT2*^*Ai14D*^*+/-*^ mouse retina. **Table S1.** List of primer sequences used for genotyping. **Table S2.** List of primer sequences used for qRT-PCR. **Movie S1. **Immunohistochemistry (IHC) staining on retinal whole mounts from a male hemizygous *Arr3*^*T2ACreERT2*^*Ai14D*^*+/-*^ mouse showed distinct tdTomato expression (red) in cone cells (left and right) labeled with green spots with the anti-ARR3 antibody (left and middle). **Movie S2.** IHC staining on a retinal crysection from a male hemizygous *Arr3*^*T2ACreERT2*^*Ai14D*^*+/-*^ mouse showed 40% of glycogen phosphorylase (GlyPh)-positive (green) cones express tdTomato (red). **Movie S3.** IHC staining for the anti-ARR3 antibody on a retinal whole mount from a female heterozygous *Arr3*^*T2ACreERT2/+*^*Ai14D*^*+/-*^ mouse showed a mosaic pattern of green (ARR3-positive cone cells, left and middle) and red (tdTomato expressing cone cells, left and right) in retinal whole mounts. It is worth noting that red and green labelled cone cells are not co-localized. **Movie S4.** IHC staining for the anti-ARR3 antibody on a retinal cryosections from a female heterozygous *Arr3*^*T2ACreERT2/+*^*Ai14D*^*+/-*^ mouse showed a mosaic pattern of green (ARR3-positive cone cells) and red (tdTomato expressing cone cells). It is worth noting that red and green labelled cone cells are not co-localized. **Movie S5.** IHC staining for PNA antibodies on a retinal cryosection from a female heterozygous *Arr3*^*T2ACreERT2/+*^*Ai14D*^*+/-*^ mouse showed around 50% of PNA-positive (green) cone cells express tdTomato (red). **Movie S6.** IHC staining on a retinal whole mount from male hemizygous *Arr3*^*P2ACreERT2*^*Ai14D*^*+/-*^ mice showed distinct tdTomato (red) expression in cone cells (left and right) with ARR3 barely labeled in green (left and middle). **Movie S7.** IHC staining on a retinal crysection from a male hemizygous *Arr3*^*P2ACreERT2*^*Ai14D*^*+/-*^ mouse showed distinct tdTomato (red) expression in cone cells with green spots labelled with ARR3 antibody. **Movie S8.** IHC staining on a retinal crysection from a male hemizygous *Arr3*^*P2ACreERT2*^*Ai14D*^*+/-*^ mouse showed 100% colocalization of GlyPh (green) and tdTomato (red). **Movie S9.** IHC staining on a retinal whole mount from a female homozygous *Arr3*^*P2ACreERT2*^*Ai14D*^*+/-*^ mouse showed a mosaic pattern of green (ARR3-positive cone cells, left and middle) and red (tdTomato expressing cone cells, left and right). It is worth noting that red and green labelled cone cells are not co-localized. **Movie S10.** IHC staining on a retinal crysection from a female homozygous *Arr3*^*P2ACreERT2*^*Ai14D*^*+/-*^ mouse showed a mosaic pattern of green (ARR3-positive cone cells) and red (tdTomato expressing cone cells). It is worth noting that red and green-labelled cone cells are not co-localized. **Dataset S1.** ERG of *Arr3*^*T2ACreERT2*^ mice at PD60. Related to Figure 4B. **Dataset S2.** SDOCT of *Arr3*^*P2ACreERT2*^ mice at PD60. Related to Figure 7B. Excel file. **Dataset S3.** ERG of *Arr3*^*P2ACreERT2*^ mice at PD4. Related to Figure 8A. Excel file. **Dataset S4.** ERG of *Arr3*^*P2ACreERT2*^ mice at PD210. Related to Figure 8B. Excel file. **Dataset S5.** Flicker ERG of *Arr3*^*P2ACreERT2*^ mice at PD40 and PD210. Related to Figure 8D, E. Excel file. **Dataset S6.** ERG of *Arr3*^*P2ACreERT2*^ mice at PD60. Related to Figure S1. Excel file

## Data Availability

Any additional data and materials are available from corresponding authors on reasonable request.
