# Supplementary material for: Spatiotemporal control of genome engineering in cone photoreceptors
Source: Cell Biosci. 2023 Jun 28;13:119. doi: 10.1186/s13578-023-01033-3 (PMC10304375; doi:10.1186/s13578-023-01033-3)
Supplement: Supplementary file 1 — Additional file 1: Figure S1. Scotopic and photopic serial intensity ERG responses from 2 month-old C57BL/6J, Arr3P2ACreERT2/+, and Arr3P2ACreERT2 mice. Figure S2. Representative Scotopic, photopic serial intensity ERG and flicker ERG frequency series responses from 10 month-old C57BL/6J and Arr3P2ACreERT2 mice. Figure S3. Cre-LoxP recombination in Gnat2CreERT2/+Ai14D+/- mice. Figure S4. Delayed induction (at 5 months old) of cone-specific Cre-LoxP recombination activity in Arr3P2ACreERT2Ai14D+/- mouse retina. Table S1. List of primer sequences used for genotyping. Table S2. List of primer sequences used for qRT-PCR. Movie S1. Immunohistochemistry (IHC) staining on retinal whole mounts from a male hemizygous Arr3T2ACreERT2Ai14D+/- mouse showed distinct tdTomato expression (red) in cone cells (left and right) labeled with green spots with the anti-ARR3 antibody (left and middle). Movie S2. IHC staining on a retinal crysection from a male hemizygous Arr3T2ACreERT2Ai14D+/- mouse showed 40% of glycogen phosphorylase (GlyPh)-positive (green) cones express tdTomato (red). Movie S3. IHC staining for the anti-ARR3 antibody on a retinal whole mount from a female heterozygous Arr3T2ACreERT2/+Ai14D+/- mouse showed a mosaic pattern of green (ARR3-positive cone cells, left and middle) and red (tdTomato expressing cone cells, left and right) in retinal whole mounts. It is worth noting that red and green labelled cone cells are not co-localized. Movie S4. IHC staining for the anti-ARR3 antibody on a retinal cryosections from a female heterozygous Arr3T2ACreERT2/+Ai14D+/- mouse showed a mosaic pattern of green (ARR3-positive cone cells) and red (tdTomato expressing cone cells). It is worth noting that red and green labelled cone cells are not co-localized. Movie S5. IHC staining for PNA antibodies on a retinal cryosection from a female heterozygous Arr3T2ACreERT2/+Ai14D+/- mouse showed around 50% of PNA-positive (green) cone cells express tdTomato (red). Movie S6. IHC staining on a [file 13578_2023_1033_MOESM1_ESM.zip › Supplementary file/Additional file-proof_1_ESM.docx]

**Supplementary Information for**

**Spatiotemporal control of genome engineering in cone photoreceptors**

Nan-Kai Wang ^1,2§†^, Pei-Kang Liu ^1,3,4,5§^, Yang Kong ^1^, Yun-Ju Tseng ^1^, Laura A. Jenny ^1^, [Nicholas D. Nolan](https://pubmed.ncbi.nlm.nih.gov/?sort=date&term=Nolan+ND&cauthor_id=34974542) ^1,6^, Nelson Chen ^1,7^, Hung-Hsi Wang ^1,8^, Chun Wei Hsu ^1^, Wan-Chun Huang ^1^, Janet R. Sparrow ^9^, Chyuan-Sheng Lin ^10^, Stephen H. Tsang ^11†^

^§^ N.-K.W. and P.-K.L. contribute equally as first authors to this work.

^†^ Corresponding authors.

**Email:**  Nan-Kai Wang ([wang.nankai@gmail.com](mailto:wang.nankai@gmail.com)); Stephen H. Tsang ([sht2@cumc.columbia.edu](mailto:sht2@cumc.columbia.edu) )

**This PDF file includes:**

Figures S1 to S4

Tables S1 to S2

Legends for Movies S1 to S10

Legends for Datasets S1 to S6

**Other supporting materials for this manuscript include the following:**

Movies S1 to S10

Datasets S1 to S6

Additional Figures

Figure S1. Scotopic and photopic serial intensity ERG responses from 2-month-old *C57BL/6J*, *Arr3^P2ACreERT2/+^*, and *Arr3^P2ACreERT2^* mice. Related to Fig. 8. At PD60, there is no statistically significant difference between the three groups in scotopic a-wave and b-wave amplitudes at different light intensities (–6, –5, –4, –3, –2, –1, 0, and 1.0 Log cd s/m^2^). In photopic ERG responses at different light intensities (–1, 0, 0.3, 0.6, 1.0, 1.48, 2.0, and 2.3 Log cd s/m^2^), the a- and b-wave amplitudes are larger in *Arr3^P2ACreERT2^* mice, however, the difference is not statistically significant. Data represent mean ± 2SE. *N* = 4, 4, and 8 for WT, *Arr3^P2ACreERT2/+^*, and *Arr3^P2ACreERT2^* mice, respectively.

**
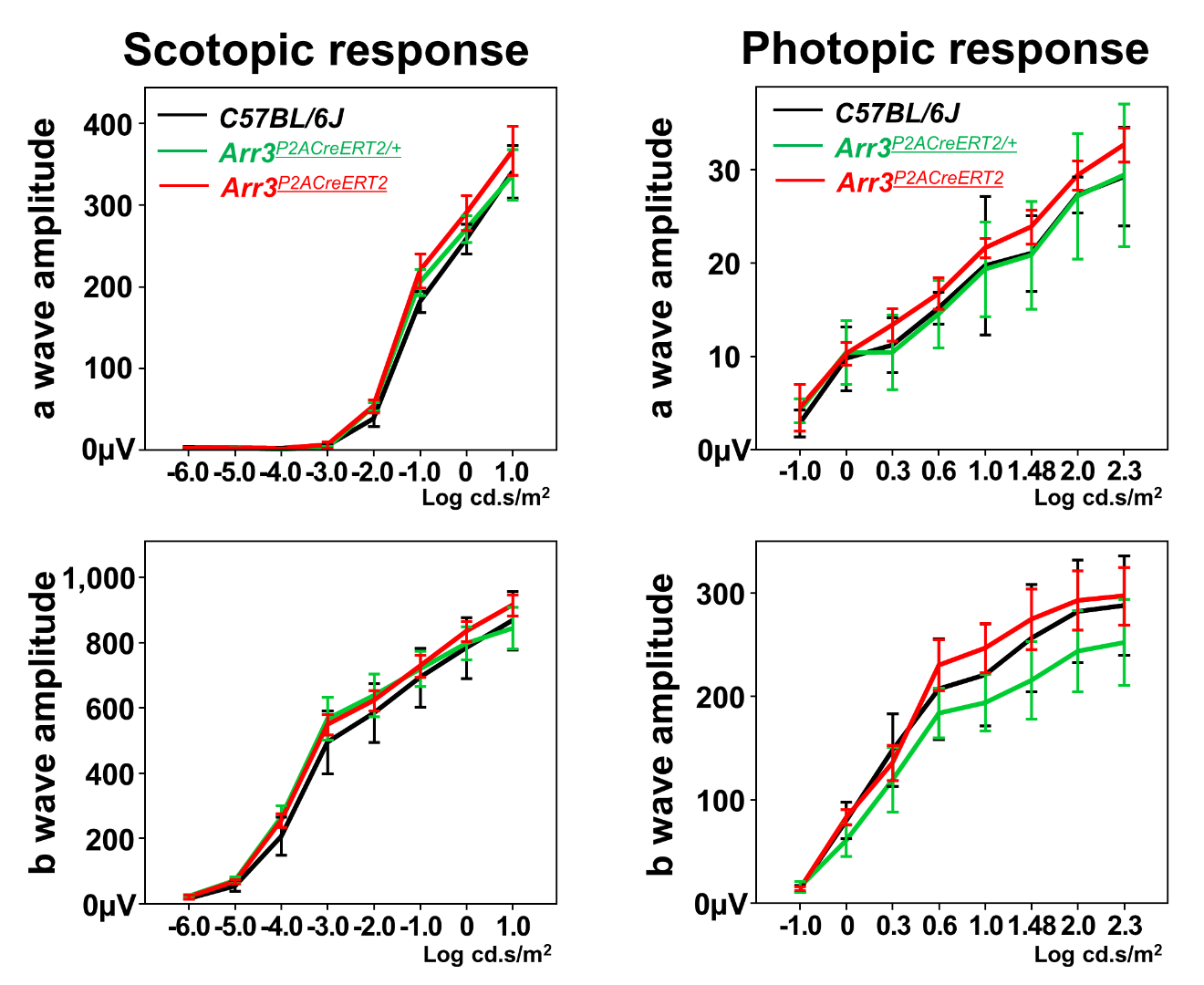
**

Figure S2. Representative Scotopic, photopic serial intensity ERG and flicker ERG frequency series responses from 10-month-old *C57BL/6J* and *Arr3^P2ACreERT2^* mice. Related to Fig. 8. At PD300, the amplitudes in scotopic, photopic serial intensities, flicker ERG frequency series are indistinguishable between WT and male hemizygous *Arr3^P2ACreERT2^* mice.


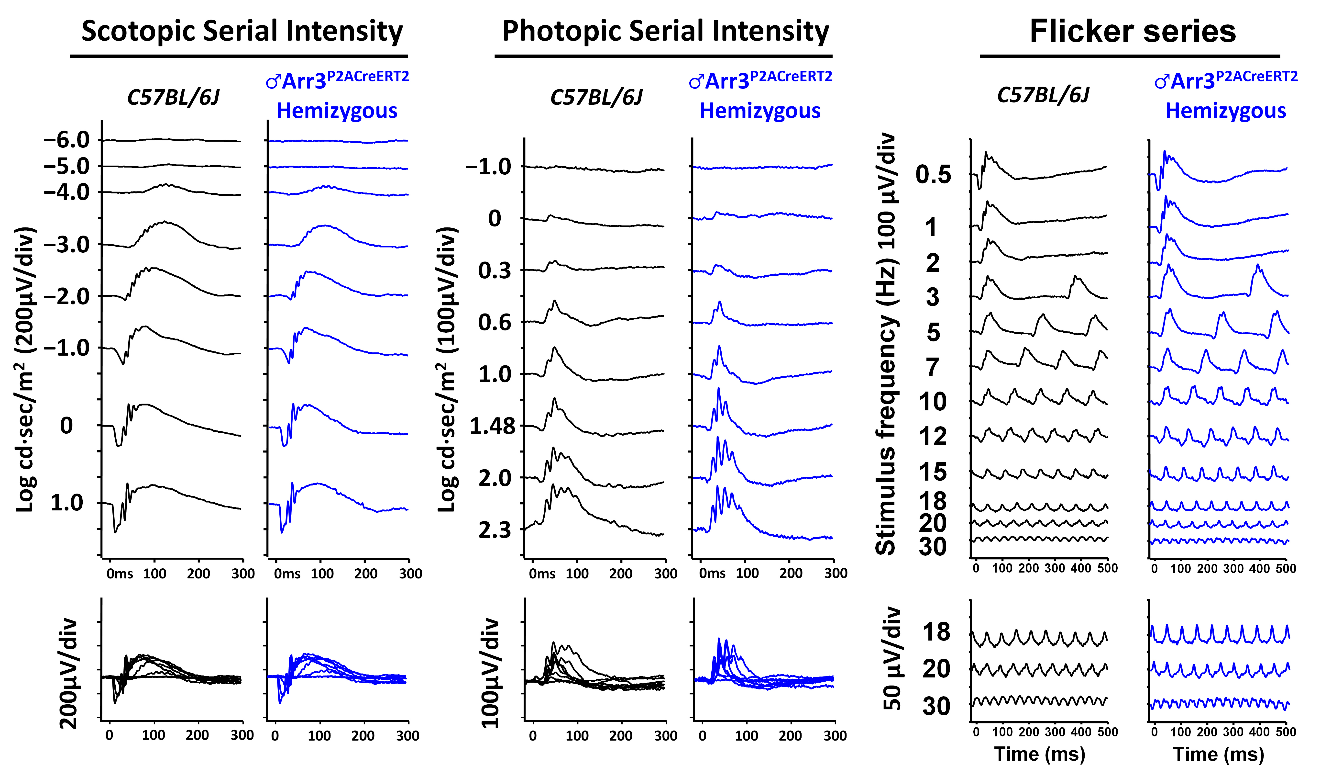


**Fig. S3. Cre-LoxP recombination in *Gnat2^CreERT2/+^Ai14D^+/-^* mice.** Immunohistochemistry (IHC) of the retinal sections from 2-month-old *Gnat2^CreERT2/+^Ai14D^+/-^* mice with an antibody against Cre recombinase (magenta) showed colocalization with td-Tomato (red). INL: inner nuclear layer; ONL: outer nuclear layer; PNA: Peanut Agglutinin; Scale bars, 20 μm.


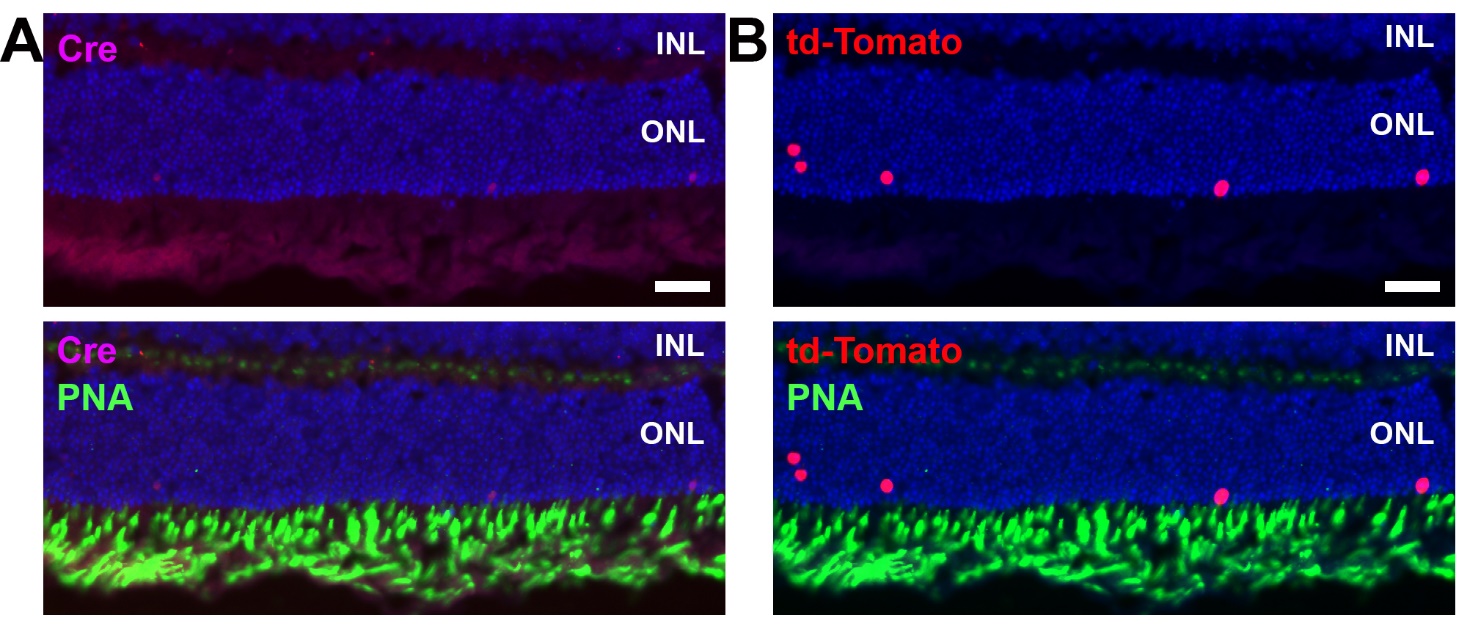


**Figure S4. Delayed induction (at 5 months old) of cone-specific Cre-LoxP recombination activity in *Arr3^P2ACreERT2^******Ai14D^+/-^* mouse retina.** (*A*) Noninvasive *in vivo* fundus SW-AF showed a homogenous dim and subsequent increased intensity in male hemizygous *Arr3^P2ACreERT2^Ai14D^+/-^* mice pre- and post-tamoxifen induction, respectively. (*B*) Immunohistochemistry (IHC) staining on cryosections from male hemizygous *Arr3^P2ACreERT2^Ai14D^+/-^* mice showed tdTomato signal in cone cells (red) did co-label with anti- glycogen phosphorylase (GlyPh) (green) [second row] and anti-PNA antibody staining (green) [third row]. Nearly 100% of GlyPh-positive cones also expressed tdTomato suggesting that the Cre-LoxP recombination efficiency was 100% efficient, with every cone cell in hemizygous *Arr3^P2ACreERT2^Ai14D^+/-^* mice expressing tdTomato. (*C-D*) IHC staining for the anti-PNA antibody on a retinal whole mount from a male hemizygous *Arr3^P2ACreERT2^Ai14D^+/-^* mouse showed a confluent, co-localized staining pattern of green (PNA-positive cone cells) and red (tdTomato expressing cone cells) in retinal whole mounts. PD: postnatal day; Tam: tamoxifen; OPL: outer plexiform layer; ONL: outer nuclear layer; IS: inner segment; Ai14D: B6.Cg-Gt(ROSA)26Sor^tm14(CAG-tdTomato)Hze^/J (JAX #007914). Scale bars, 200 μm in *A*, 20 μm in *B* and *D*, and 1000 μm in *C*.

**
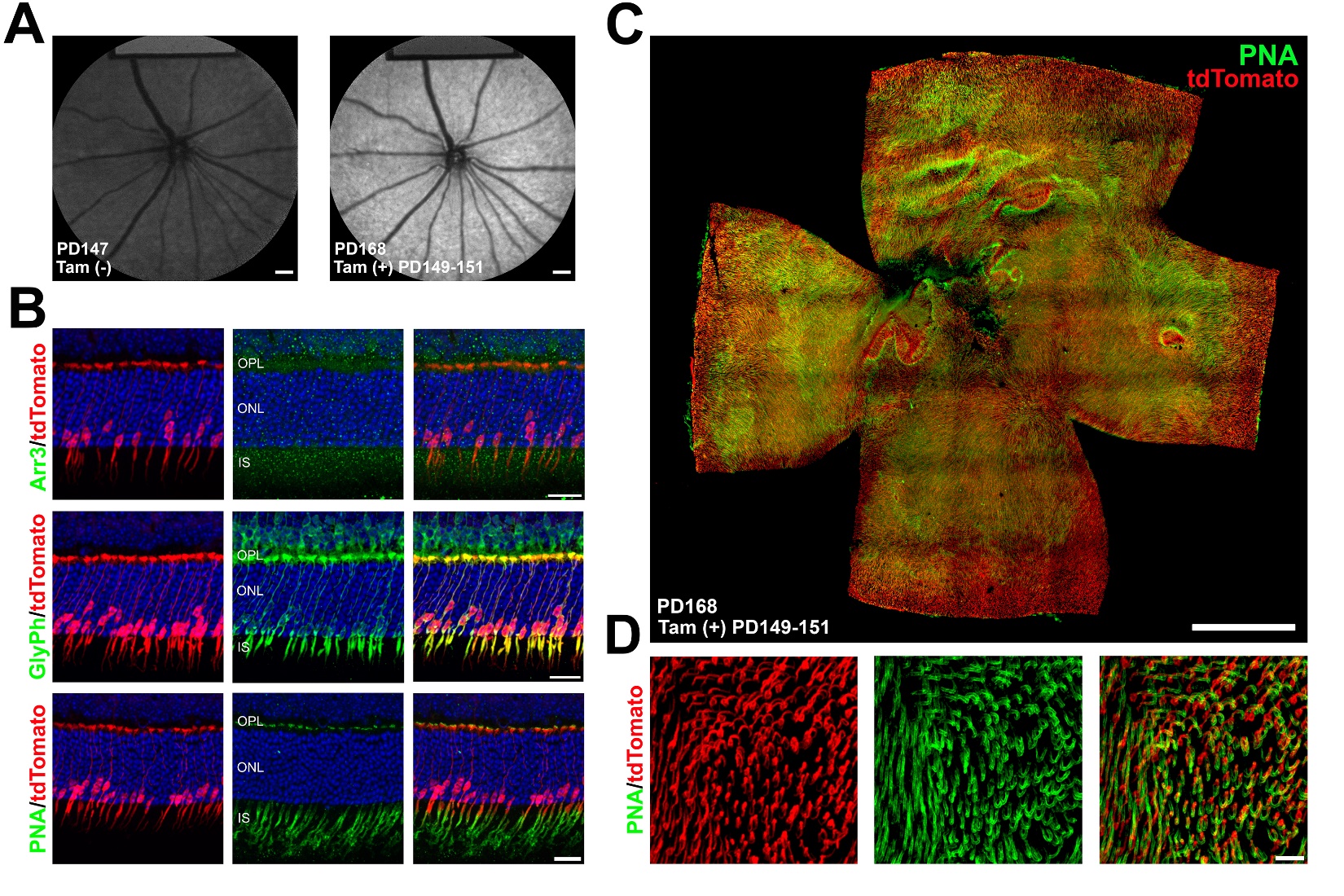
**

Additional Tables

Table S1. List of primer sequences used for genotyping

| **Gene name** |  | **Primer sequence (5’ – 3’)** | **Product size (bp)** |
| --- | --- | --- | --- |
| *Gnat2 (Knock in)* | Forward | ATTCCGCTAGACATAAAGGGGACAA | 375 |
|  | Reverse | ACGGACAGAAGCATTTTCCAGGTAT |  |
| *Arr3 (Wild type)* | Forward | F1: GAAGCAGGGAAGGGATTACC | 437 |
|  | Reverse | R2: AGGGTAGCTGAGGTCAGACAGAAAA |  |
| *Arr3 (Knock in)* | Forward | F1: GAAGCAGGGAAGGGATTACC | 254 |
|  | Reverse | R1: AGGCAAATTTTGGTGTACGG |  |

Table S2. List of primer sequences used for qRT-PCR

| **Gene name** |  | **Primer sequence (5’ – 3’)** |
| --- | --- | --- |
| *Gapdh* | Forward | AATGGTGAAGGTCGGTGTG |
|  | Reverse | CTGGAAGATGGTGATGGGC |
| *Arr3* | Forward | AGGTCGAAAGTTGTTTGTCAGG |
|  | Reverse | TAGGCATTGACCCCGAGCTT |
| *Opn1mw* | Forward | AGCACCAAAGGTCCCTTTGAA |
|  | Reverse | GGTCAGCAACTGCCAAGTTCA |
| *Cnga3* | Forward | CCCCGACCCAACTTTCAATA |
|  | Reverse | TTCATCGTGTAAGTGCCTGG |

Additional legends for Movies

Movie S1.

Immunohistochemistry (IHC) staining on retinal whole mounts from a male hemizygous *Arr3^T2ACreERT2^Ai14D^+/-^* mouse showed distinct tdTomato expression (red) in cone cells (left and right) labeled with green spots with the anti-ARR3 antibody (left and middle).

Movie S2.

IHC staining on a retinal crysection from a male hemizygous *Arr3^T2ACreERT2^Ai14D^+/-^* mouse showed 40% of glycogen phosphorylase (GlyPh)-positive (green) cones express tdTomato (red).

Movie S3.

IHC staining for the anti-ARR3 antibody on a retinal whole mount from a female heterozygous *Arr3^T2ACreERT2/+^Ai14D^+/-^* mouse showed a mosaic pattern of green (ARR3-positive cone cells, left and middle) and red (tdTomato expressing cone cells, left and right) in retinal whole mounts. It is worth noting that red and green labelled cone cells are not co-localized.

Movie S4.

IHC staining for the anti-ARR3 antibody on a retinal cryosections from a female heterozygous *Arr3^T2ACreERT2/+^Ai14D^+/-^* mouse showed a mosaic pattern of green (ARR3-positive cone cells) and red (tdTomato expressing cone cells). It is worth noting that red and green labelled cone cells are not co-localized.

Movie S5.

IHC staining for PNA antibodies on a retinal cryosection from a female heterozygous *Arr3^T2ACreERT2/+^Ai14D^+/-^* mouse showed around 50% of PNA-positive (green) cone cells express tdTomato (red).

Movie S6.

IHC staining on a retinal whole mount from male hemizygous *Arr3^P2ACreERT2^Ai14D^+/-^* mice showed distinct tdTomato (red) expression in cone cells (left and right) with ARR3 barely labeled in green (left and middle).

Movie S7.

IHC staining on a retinal crysection from a male hemizygous *Arr3^P2ACreERT2^Ai14D^+/-^* mouse showed distinct tdTomato (red) expression in cone cells with green spots labelled with ARR3 antibody.

Movie S8.

IHC staining on a retinal crysection from a male hemizygous *Arr3^P2ACreERT2^Ai14D^+/-^* mouse showed 100% colocalization of GlyPh (green) and tdTomato (red).

Movie S9.

IHC staining on a retinal whole mount from a female homozygous *Arr3^P2ACreERT2^Ai14D^+/-^* mouse showed a mosaic pattern of green (ARR3-positive cone cells, left and middle) and red (tdTomato expressing cone cells, left and right). It is worth noting that red and green labelled cone cells are not co-localized.

Movie S10.

IHC staining on a retinal crysection from a female homozygous *Arr3^P2ACreERT2^Ai14D^+/-^* mouse showed a mosaic pattern of green (ARR3-positive cone cells) and red (tdTomato expressing cone cells). It is worth noting that red and green-labelled cone cells are not co-localized.

Additional legends for Datasets

Dataset S1. ERG of *Arr3^T2ACreERT2^* mice at PD60. Related to Figure 4B.

Excel file

Dataset S2. SDOCT of *Arr3^P2ACreERT2^* mice at PD60. Related to Figure 7B.

Excel file

Dataset S3. ERG of *Arr3^P2ACreERT2^* mice at PD4. Related to Figure 8A.

Excel file

Dataset S4. ERG of *Arr3^P2ACreERT2^* mice at PD210. Related to Figure 8B.

Excel file

Dataset S5. Flicker ERG of *Arr3^P2ACreERT2^* mice at PD40 and PD210. Related to Figure 8D and E.

Excel file

Dataset S6. ERG of *Arr3^P2ACreERT2^* mice at PD60. Related to Figure S1.

Excel file
