# Supplementary figures and images for: Spatiotemporal control of genome engineering in cone photoreceptors
Source: Cell Biosci. 2023 Jun 28;13:119. doi: 10.1186/s13578-023-01033-3 (PMC10304375; doi:10.1186/s13578-023-01033-3)

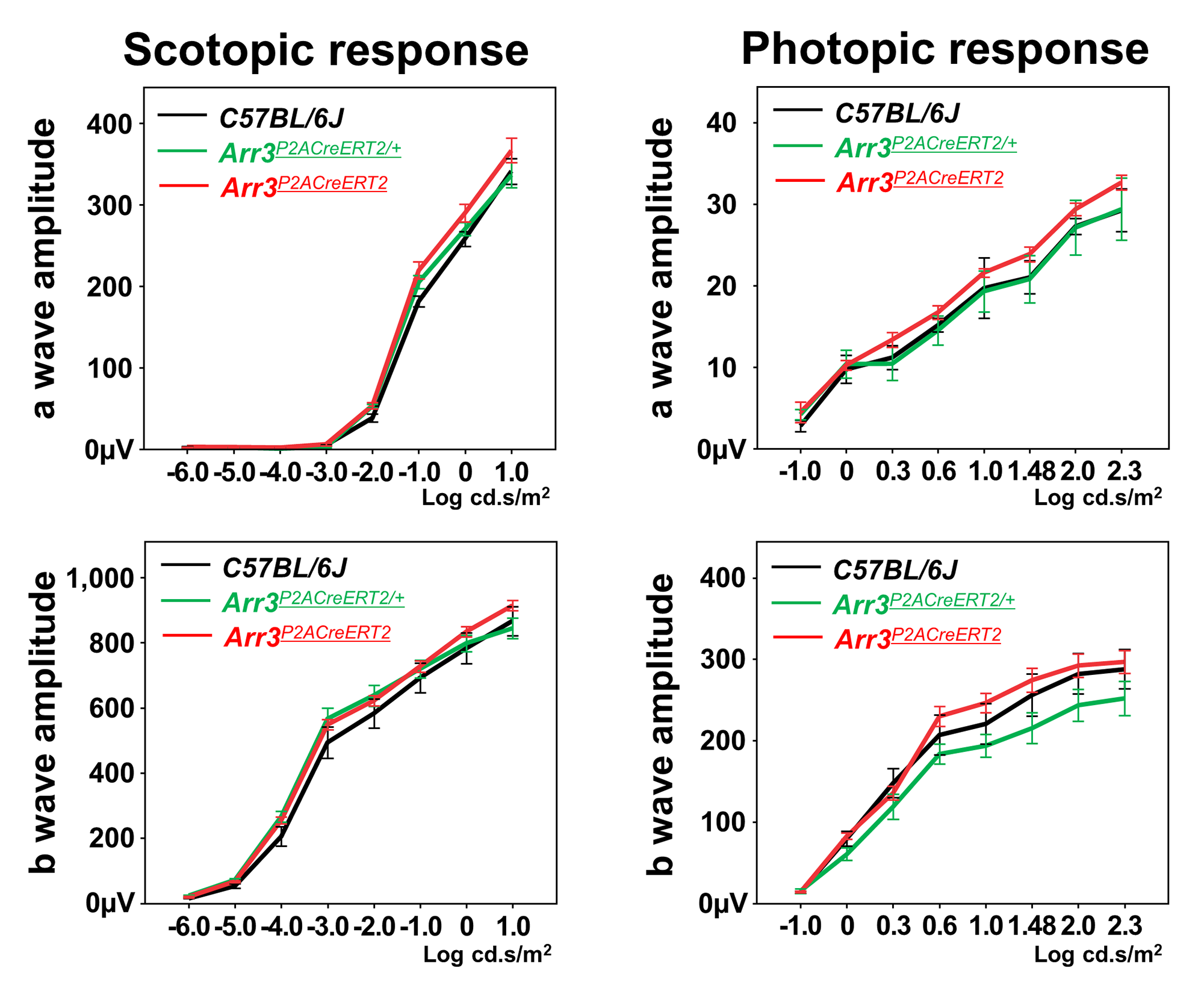

Supplement: Supplementary file 1 — Additional file 1: Figure S1. Scotopic and photopic serial intensity ERG responses from 2 month-old C57BL/6J, Arr3P2ACreERT2/+, and Arr3P2ACreERT2 mice. Figure S2. Representative Scotopic, photopic serial intensity ERG and flicker ERG frequency series responses from 10 month-old C57BL/6J and Arr3P2ACreERT2 mice. Figure S3. Cre-LoxP recombination in Gnat2CreERT2/+Ai14D+/- mice. Figure S4. Delayed induction (at 5 months old) of cone-specific Cre-LoxP recombination activity in Arr3P2ACreERT2Ai14D+/- mouse retina. Table S1. List of primer sequences used for genotyping. Table S2. List of primer sequences used for qRT-PCR. Movie S1. Immunohistochemistry (IHC) staining on retinal whole mounts from a male hemizygous Arr3T2ACreERT2Ai14D+/- mouse showed distinct tdTomato expression (red) in cone cells (left and right) labeled with green spots with the anti-ARR3 antibody (left and middle). Movie S2. IHC staining on a retinal crysection from a male hemizygous Arr3T2ACreERT2Ai14D+/- mouse showed 40% of glycogen phosphorylase (GlyPh)-positive (green) cones express tdTomato (red). Movie S3. IHC staining for the anti-ARR3 antibody on a retinal whole mount from a female heterozygous Arr3T2ACreERT2/+Ai14D+/- mouse showed a mosaic pattern of green (ARR3-positive cone cells, left and middle) and red (tdTomato expressing cone cells, left and right) in retinal whole mounts. It is worth noting that red and green labelled cone cells are not co-localized. Movie S4. IHC staining for the anti-ARR3 antibody on a retinal cryosections from a female heterozygous Arr3T2ACreERT2/+Ai14D+/- mouse showed a mosaic pattern of green (ARR3-positive cone cells) and red (tdTomato expressing cone cells). It is worth noting that red and green labelled cone cells are not co-localized. Movie S5. IHC staining for PNA antibodies on a retinal cryosection from a female heterozygous Arr3T2ACreERT2/+Ai14D+/- mouse showed around 50% of PNA-positive (green) cone cells express tdTomato (red). Movie S6. IHC staining on a [file 13578_2023_1033_MOESM1_ESM.zip › Supplementary file/Figure S1.tif]

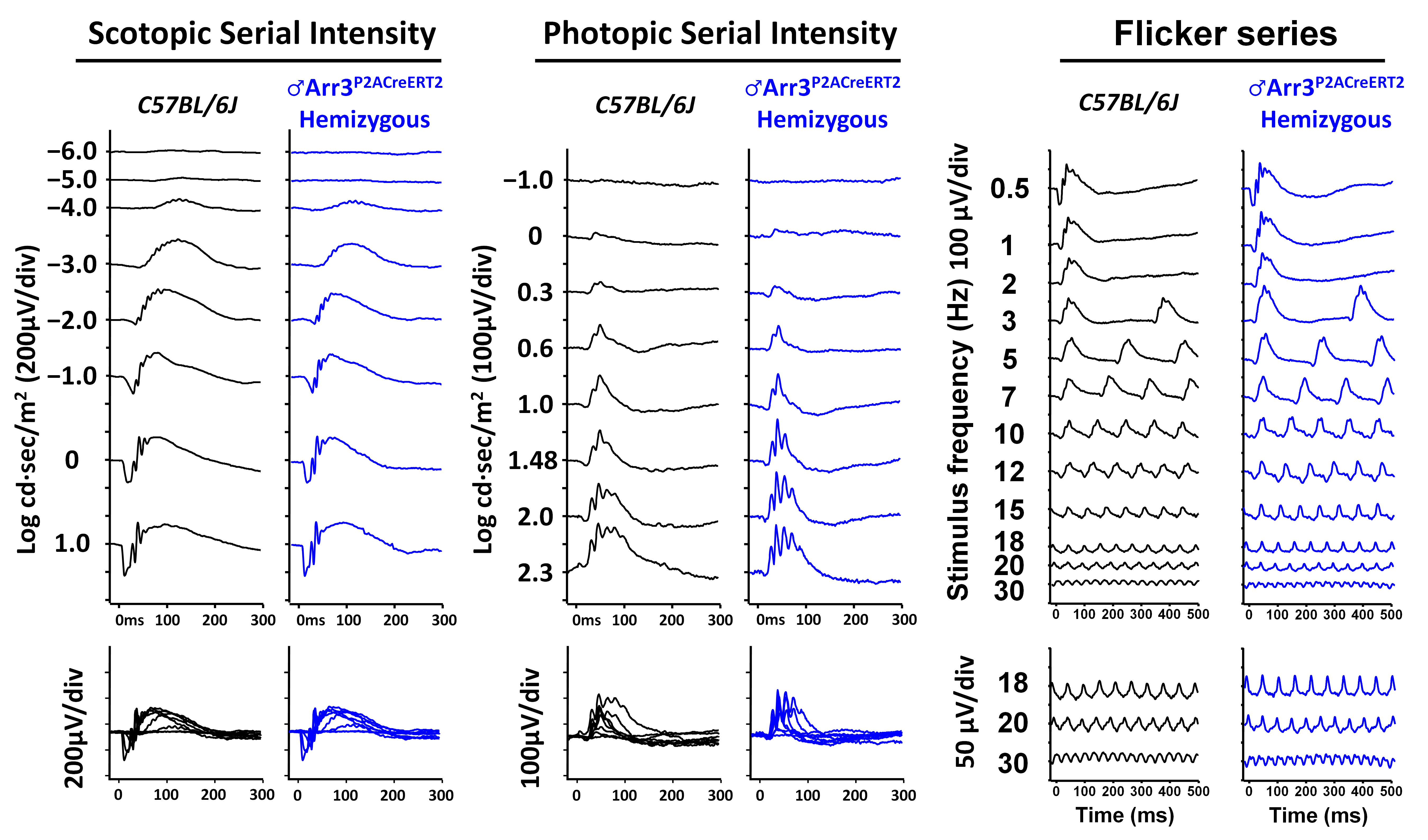

Supplement: Supplementary file 1 — Additional file 1: Figure S1. Scotopic and photopic serial intensity ERG responses from 2 month-old C57BL/6J, Arr3P2ACreERT2/+, and Arr3P2ACreERT2 mice. Figure S2. Representative Scotopic, photopic serial intensity ERG and flicker ERG frequency series responses from 10 month-old C57BL/6J and Arr3P2ACreERT2 mice. Figure S3. Cre-LoxP recombination in Gnat2CreERT2/+Ai14D+/- mice. Figure S4. Delayed induction (at 5 months old) of cone-specific Cre-LoxP recombination activity in Arr3P2ACreERT2Ai14D+/- mouse retina. Table S1. List of primer sequences used for genotyping. Table S2. List of primer sequences used for qRT-PCR. Movie S1. Immunohistochemistry (IHC) staining on retinal whole mounts from a male hemizygous Arr3T2ACreERT2Ai14D+/- mouse showed distinct tdTomato expression (red) in cone cells (left and right) labeled with green spots with the anti-ARR3 antibody (left and middle). Movie S2. IHC staining on a retinal crysection from a male hemizygous Arr3T2ACreERT2Ai14D+/- mouse showed 40% of glycogen phosphorylase (GlyPh)-positive (green) cones express tdTomato (red). Movie S3. IHC staining for the anti-ARR3 antibody on a retinal whole mount from a female heterozygous Arr3T2ACreERT2/+Ai14D+/- mouse showed a mosaic pattern of green (ARR3-positive cone cells, left and middle) and red (tdTomato expressing cone cells, left and right) in retinal whole mounts. It is worth noting that red and green labelled cone cells are not co-localized. Movie S4. IHC staining for the anti-ARR3 antibody on a retinal cryosections from a female heterozygous Arr3T2ACreERT2/+Ai14D+/- mouse showed a mosaic pattern of green (ARR3-positive cone cells) and red (tdTomato expressing cone cells). It is worth noting that red and green labelled cone cells are not co-localized. Movie S5. IHC staining for PNA antibodies on a retinal cryosection from a female heterozygous Arr3T2ACreERT2/+Ai14D+/- mouse showed around 50% of PNA-positive (green) cone cells express tdTomato (red). Movie S6. IHC staining on a [file 13578_2023_1033_MOESM1_ESM.zip › Supplementary file/Figure S2.jpg]

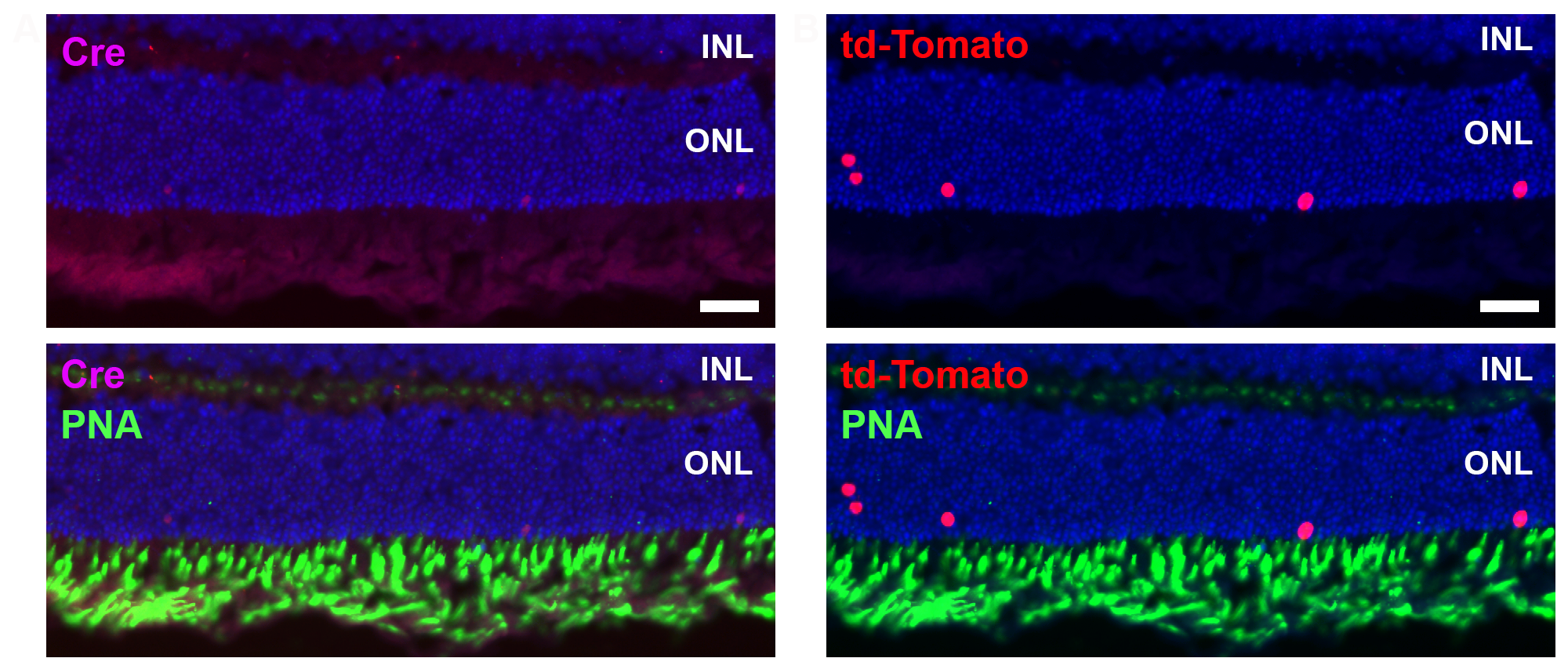

Supplement: Supplementary file 1 — Additional file 1: Figure S1. Scotopic and photopic serial intensity ERG responses from 2 month-old C57BL/6J, Arr3P2ACreERT2/+, and Arr3P2ACreERT2 mice. Figure S2. Representative Scotopic, photopic serial intensity ERG and flicker ERG frequency series responses from 10 month-old C57BL/6J and Arr3P2ACreERT2 mice. Figure S3. Cre-LoxP recombination in Gnat2CreERT2/+Ai14D+/- mice. Figure S4. Delayed induction (at 5 months old) of cone-specific Cre-LoxP recombination activity in Arr3P2ACreERT2Ai14D+/- mouse retina. Table S1. List of primer sequences used for genotyping. Table S2. List of primer sequences used for qRT-PCR. Movie S1. Immunohistochemistry (IHC) staining on retinal whole mounts from a male hemizygous Arr3T2ACreERT2Ai14D+/- mouse showed distinct tdTomato expression (red) in cone cells (left and right) labeled with green spots with the anti-ARR3 antibody (left and middle). Movie S2. IHC staining on a retinal crysection from a male hemizygous Arr3T2ACreERT2Ai14D+/- mouse showed 40% of glycogen phosphorylase (GlyPh)-positive (green) cones express tdTomato (red). Movie S3. IHC staining for the anti-ARR3 antibody on a retinal whole mount from a female heterozygous Arr3T2ACreERT2/+Ai14D+/- mouse showed a mosaic pattern of green (ARR3-positive cone cells, left and middle) and red (tdTomato expressing cone cells, left and right) in retinal whole mounts. It is worth noting that red and green labelled cone cells are not co-localized. Movie S4. IHC staining for the anti-ARR3 antibody on a retinal cryosections from a female heterozygous Arr3T2ACreERT2/+Ai14D+/- mouse showed a mosaic pattern of green (ARR3-positive cone cells) and red (tdTomato expressing cone cells). It is worth noting that red and green labelled cone cells are not co-localized. Movie S5. IHC staining for PNA antibodies on a retinal cryosection from a female heterozygous Arr3T2ACreERT2/+Ai14D+/- mouse showed around 50% of PNA-positive (green) cone cells express tdTomato (red). Movie S6. IHC staining on a [file 13578_2023_1033_MOESM1_ESM.zip › Supplementary file/Figure S3.tif]

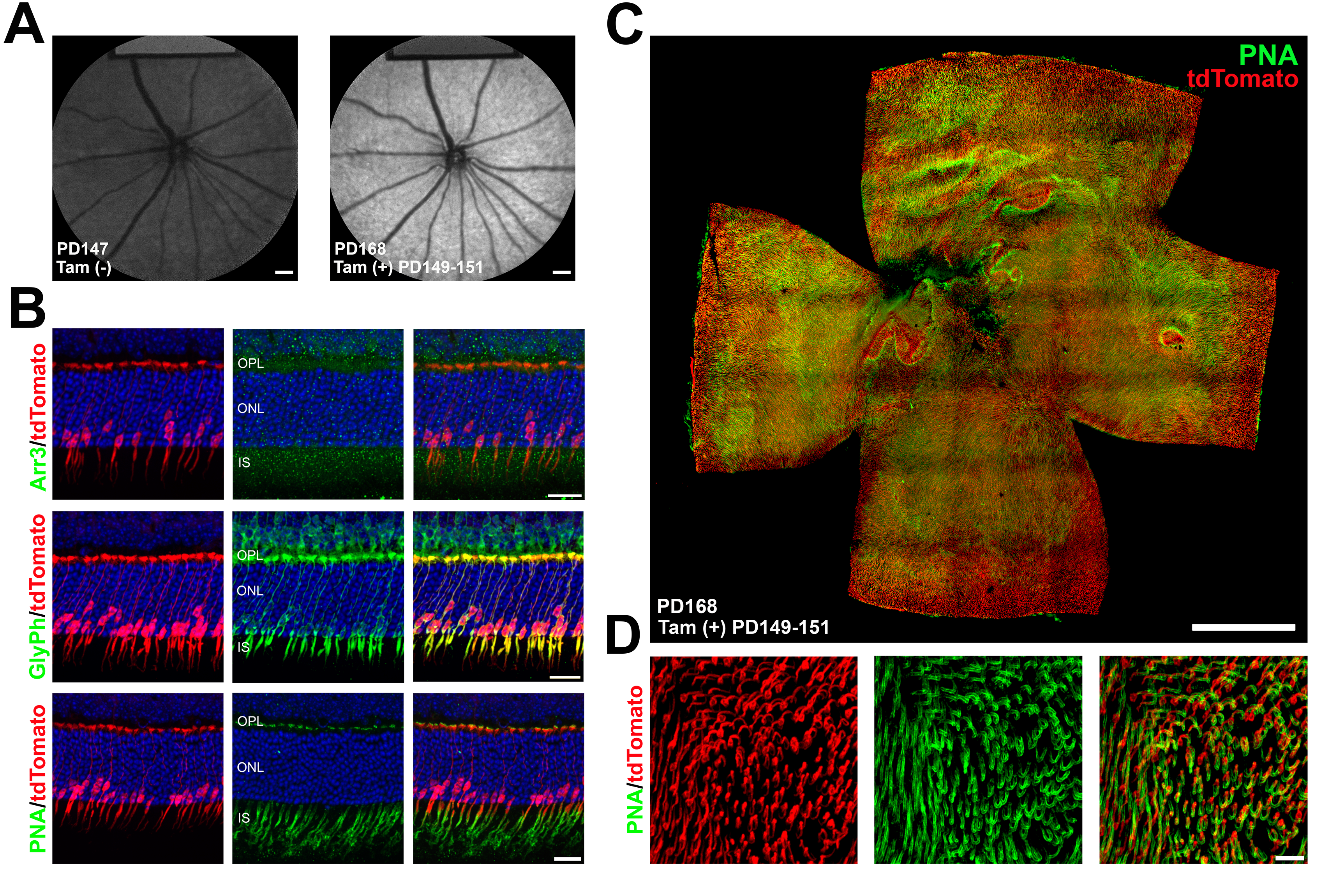

Supplement: Supplementary file 1 — Additional file 1: Figure S1. Scotopic and photopic serial intensity ERG responses from 2 month-old C57BL/6J, Arr3P2ACreERT2/+, and Arr3P2ACreERT2 mice. Figure S2. Representative Scotopic, photopic serial intensity ERG and flicker ERG frequency series responses from 10 month-old C57BL/6J and Arr3P2ACreERT2 mice. Figure S3. Cre-LoxP recombination in Gnat2CreERT2/+Ai14D+/- mice. Figure S4. Delayed induction (at 5 months old) of cone-specific Cre-LoxP recombination activity in Arr3P2ACreERT2Ai14D+/- mouse retina. Table S1. List of primer sequences used for genotyping. Table S2. List of primer sequences used for qRT-PCR. Movie S1. Immunohistochemistry (IHC) staining on retinal whole mounts from a male hemizygous Arr3T2ACreERT2Ai14D+/- mouse showed distinct tdTomato expression (red) in cone cells (left and right) labeled with green spots with the anti-ARR3 antibody (left and middle). Movie S2. IHC staining on a retinal crysection from a male hemizygous Arr3T2ACreERT2Ai14D+/- mouse showed 40% of glycogen phosphorylase (GlyPh)-positive (green) cones express tdTomato (red). Movie S3. IHC staining for the anti-ARR3 antibody on a retinal whole mount from a female heterozygous Arr3T2ACreERT2/+Ai14D+/- mouse showed a mosaic pattern of green (ARR3-positive cone cells, left and middle) and red (tdTomato expressing cone cells, left and right) in retinal whole mounts. It is worth noting that red and green labelled cone cells are not co-localized. Movie S4. IHC staining for the anti-ARR3 antibody on a retinal cryosections from a female heterozygous Arr3T2ACreERT2/+Ai14D+/- mouse showed a mosaic pattern of green (ARR3-positive cone cells) and red (tdTomato expressing cone cells). It is worth noting that red and green labelled cone cells are not co-localized. Movie S5. IHC staining for PNA antibodies on a retinal cryosection from a female heterozygous Arr3T2ACreERT2/+Ai14D+/- mouse showed around 50% of PNA-positive (green) cone cells express tdTomato (red). Movie S6. IHC staining on a [file 13578_2023_1033_MOESM1_ESM.zip › Supplementary file/Figure S4 small.tif]
